# Identification of a bacterial NCS1 family transporter enabling high-affinity uptake of the antidiabetic drug metformin

**DOI:** 10.1128/aem.02306-25

**Published:** 2026-06-12

**Authors:** Zhi-Jing Xu, Tao Li, Ning-Yi Zhou

**Affiliations:** 1 State Key Laboratory of Microbial Metabolism, Joint International Research Laboratory of Metabolic and Developmental Sciences, and School of Life Sciences and Biotechnology, Shanghai Jiao Tong University 12474, Shanghai, China; Washington University in St Louis, St. Louis, Missouri, USA

**Keywords:** biodegradation, metformin, nucleobase cation symporter 1, transport

## Abstract

Metformin is a first-line antidiabetic medication for type 2 diabetes and a widely dispersed emerging pollutant in aquatic systems. During medication, its strong positive charge (pKa  =  12.4) under physiological conditions necessitates cellular uptake via non-specific cationic transporters, such as organic cation transporter 1 (OCT1) in humans. During bacterial biodegradation of metformin, it was also proposed that the transport of metformin into bacterial cells is a prerequisite. Despite progress in elucidating the biodegradation mechanism of metformin, the bacterial membrane transport systems involved remain largely unexplored. Here, we characterize a metformin transporter (MetT) from the metformin utilizer *Aminobacter* sp. strain NyZ550. Gene knockout and complementation experiments demonstrate that *metT* is essential for the growth of strain NyZ550 on metformin. Efficient bacterial degradation of metformin necessitates the presence of both the membrane transporter and the downstream catabolic enzyme. Phylogenetic analysis revealed that MetT is a member of the nucleobase cation symporter 1 (NCS1) family, but forms a separate clade distinct from previously characterized NCS1 members. It exhibits distinct polar localization within the cytoplasmic membrane, as evidenced by a MetT-green fluorescent protein fusion construct. Radiolabeled uptake assays using ^14^C-metformin revealed a substrate transport affinity (*K*_m_) of 15.90 ± 1.75 µM, which starkly contrasts with the millimolar-range *K*_m_ of human OCT1. Structural modeling and site-directed mutagenesis revealed a substrate-binding cavity consisting of aromatic residues, likely facilitating substrate recognition through cation–π interactions. Overall, this study characterizes a functional bacterial metformin transporter and a new member from the emerging NCS1 transporter family.

**IMPORTANCE:**

The increasing global consumption of pharmaceuticals worldwide is contributing to the release of drugs and their catabolic byproducts into ecological systems through various routes, posing significant threats to environmental stability and public health. Environmental microbes can metabolize many of these molecules by evolving specific enzymes; however, efficient degradation necessitates their transport across the cell membrane, a process that remains poorly understood. In this study, we identified metformin transporter (MetT), a bacterial metformin transporter, from the metformin-utilizing strain NyZ550. MetT exhibits distinct specificity and transport affinity compared to human metformin transporters. This discovery offers new insights into the transport of metformin in prokaryotic cells and opens new avenues for exploring drug–microbe interactions and their implications for environmental biodegradation and gut biology.

## INTRODUCTION

As global pharmaceutical consumption rises, a significant amount of drugs that are not fully absorbed or metabolized by the human body are ultimately excreted in urine or feces and then enter wastewater treatment plants (WWTPs) (1, 2). These active pharmaceutical ingredients (APIs) are increasingly being discharged into aquatic ecosystems through WWTPs’ effluents, with environmental concentrations ranging from ng/L to μg/L levels (3). Microbial degradation plays a crucial role in the environmental remediation of APIs. Particularly, bacteria have evolved sophisticated systems enabling them to assimilate and catabolize APIs as nutrient sources (4). The biochemical pathways and enzymatic mechanisms for the biodegradation of many environmentally occurring APIs, such as metformin, have been extensively investigated. However, efficient bacterial degradation also depends on the uptake of these extracellular compounds, making them accessible to intracellular catabolic enzymes (5, 6). Despite its importance, the mechanisms by which APIs are transported into bacterial cells via membrane transport systems remain largely unknown.

Metformin (1,1-dimethylbiguanide) has been a widely used oral antihyperglycemic medication for the treatment of type 2 diabetes (T2D) for more than six decades (7). Recent studies have indicated that metformin provides health benefits extending beyond the management of type 2 diabetes, encompassing anti-obesity effects (8), anti-aging properties (9), and anti-tumor activities (10). Upon oral administration, metformin is initially absorbed by intestinal enterocytes and subsequently enters the hepatic portal system, delivering it to the liver (11). With a dissociation constant (pKa) of 12.4, metformin exists as a hydrophilic monoprotonated cation under physiological pH conditions (12, 13). Consequently, the pharmacodynamics of metformin are strictly dependent on transporters to overcome its poor permeability, including OCT1–3 (*SLC22A1–3*) (14), plasma membrane monoamine transporter PMAT (*SLC29A4*) (15), multidrug and toxin extrusion protein MATE1 (*SLC47A1*) and MATE2-K (*SLC47A2*) (16). Genetic polymorphisms within these transporters have been associated with individual differences in metformin efficacy and its side effects, underscoring their clinical significance (17, 18). Gut microbes can also influence the efficacy of metformin (19); however, the mechanisms by which they absorb and respond to metformin are not well understood.

Currently, metformin is taken daily by over 250 million people worldwide at doses ranging from 0.5 to 2.5 grams, and it is excreted in an unchanged form, leading to its environmental accumulation (7, 20). Biodegradation is a natural process in which microorganisms can effectively break down organic pollutants. Recent genomic and functional analyses have identified several metformin-degrading bacterial strains belonging to the genera *Aminobacter* and *Pseudomonas*, which share a conserved catabolic pathway initiated by a unique metformin hydrolase (21–23). Given the high pKa value of metformin, the efficient utilization of metformin by these strains necessitates not only intracellular catabolic enzymes but also a dedicated transport system to facilitate its passage across the bacterial cell envelope (24). However, genomic analyses indicate that there are no homologs of known human-derived metformin transporters in these metformin-degrading bacterial genomes. This strongly suggests the potential existence of a phylogenetically distinct metformin uptake system in bacteria. Therefore, the molecular mechanisms underlying the microbial uptake and metabolism of metformin, as well as their physiological implications, require further sophisticated investigation.

The nucleobase cation symporter 1 (NCS1) family proteins are membrane transporters that are widely found in bacteria, archaea, fungi, and plants (25). NCS1 transporters utilize proton (H^+^) or sodium (Na^+^) gradients to drive the inward cellular transport of nucleobases, nucleosides, hydantoin, and their analogs (26). NCS1 family members are characterized by a conserved structural architecture, featuring 12 transmembrane α-helical domains. The arrangement features a pseudosymmetric structure comprising five transmembrane helices and an inverted repeat. Here, the two repeating units intertwine to create two distinct domains (27). In contrast to the growing number of annotated NCS1 proteins in databases, only a few have been documented for their functions and structures. They include the hydantoin transporter Mhp1 from *Mycobacterium liquefaciens* (28–31) and the cytosine transporter CodB from *Pseudomonas aeruginosa* (32). Other experimentally characterized bacterial NCS1 family proteins are from *Bacillus subtilis* (33), *Escherichia coli* (34), and *Pseudomonas aeruginosa* (35), and they share the common feature of transporting substrates containing nitrogenous heterocyclic rings. In addition to the internalization of these compounds for use as intermediates of nucleotide biosynthesis or sources of carbon and nitrogen, it remains unknown whether NCS1 family proteins also facilitate the uptake of other compounds.

Here, we identified an NCS1 family protein MetT, capable of facilitating the transport of metformin in a metformin-utilizing bacterium, *Aminobacter* sp. strain NyZ550. MetT displays a high substrate specificity for metformin, with a *K*_m_ value of 15.90 ± 1.75 µM. The low amino acid sequence identity with previously characterized NCS1 family proteins and its independent clade within a phylogenetic tree suggest that MetT represents a novel NCS1 family transporter evolved to recognize synthetic metformin without heterocyclic rings. Our findings expand the understanding of the functional diversity among NCS1 family transporters.

## RESULTS

### Deletion of *metT* eliminates the growth ability of strain NyZ550 on metformin

**Fig 1:**
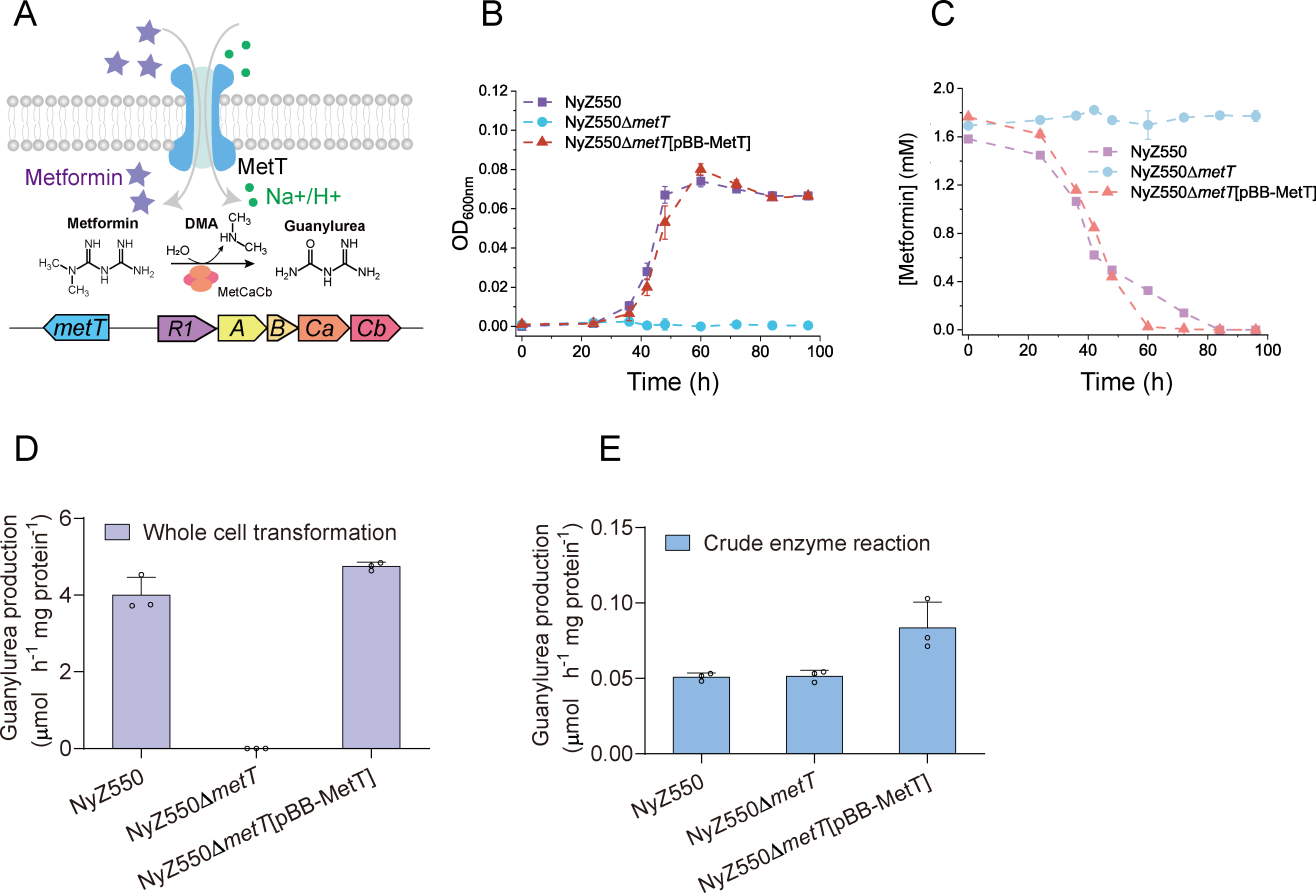
The process of growth and degradation of metformin is strictly dependent on MetT, by using strains NyZ550, NyZ550Δ*metT,* and NyZ550Δ*metT*[pBB-MetT]. (**A**) Schematic representation of the metformin transporter and the genes involved. (**B**) Growth curves on metformin as a sole source of carbon, nitrogen, and energy. (**C**) Metformin degradation by strain NyZ550 and its derivatives. (**D**) The production of guanylurea in whole-cell transformation assay using metformin as substrate. (**E**) The production of guanylurea in crude enzyme using metformin as substrate. In both whole-cell transformation (**D**) and crude enzyme assays (**E**), the guanylurea production is quantified as the micromoles of guanylurea generated per milligram of total protein per hour (μmol h^−1^ mg protein^−1^). Data are presented as the mean ± standard deviation (SD) from three independent biological replicates.

In the recently characterized metformin degrader *Aminobacter* sp. strain NyZ550, the genes encoding a Ni^2+^-dependent metformin hydrolase (MetCaCb) are located within a gene cluster that also contains genes encoding putative nickel incorporation-associated proteins (MetAB), a transcriptional regulator (MetR1), and a transporter (MetT). The gene cluster *metTR1ABCaCb* is highly conserved among all reported metformin-utilizing strains (Fig. 1A; Fig. S1). The knockout of either *metCaCb* or *metAB* eliminated the strain’s capability of growing on metformin (21), indicating the necessity of these genes for the utilization of metformin. Although the specific role of the NCS1 family transporter MetT in the metformin metabolism was not previously known, quantitative real-time PCR (RT-qPCR) analysis in this study revealed that *metT* expression was significantly upregulated (a 7.66-fold increase at 24 h) under metformin stress (Fig. S2), suggesting its potential involvement in the degradation process.

To investigate the function of MetT, we constructed a *metT*-deleted mutant NyZ550Δ*metT* and a complemented strain NyZ550Δ*metT*[pBB-MetT]. Growth assays using metformin as the sole source of carbon, nitrogen, and energy demonstrated that the strain NyZ550Δ*metT* had completely lost the capability of growing on metformin (Fig. 1B). This was consistent with the negligible consumption of metformin observed in the culture medium with NyZ550Δ*metT* (Fig. 1C). In contrast, the complemented strain NyZ550Δ*metT*[pBB-MetT] restored the ability to utilize metformin for growth (Fig. 1B). Moreover, even when the metformin concentration was increased from 0 to 10 mM, strain NyZ550Δ*metT* remained unable to grow on metformin, indicating that metformin uptake in strain NyZ550 is strictly dependent on MetT-mediated transport and cannot be compensated by alternative nonspecific transport systems or passive diffusion (Fig. S3). As a control, no difference was observed among the wild-type (WT), *metT* knockout, and complemented strains (Fig. S4) in growth assays using glucose as the sole carbon source

To determine whether the growth deficiency of strain NyZ550Δ*metT* on metformin results from transporter-mediated restriction of intracellular metformin rather than impaired catabolic enzymes, we compared the metformin degradation capacities of the WT strain and mutant strains by transformation assays using whole cells or cell lysates. A strategy based on mass spectrometry was used to quantify guanylurea, a product from metformin hydrolysis. Whole-cell biotransformation assay showed that guanylurea (*m*/*z* 103) was produced by both strain NyZ550 and the complemented strain NyZ550Δ*metT*[pBB-MetT] (Fig. S5), with rates of 4.0 and 4.75 μmol h^−1^ mg protein^−1^, respectively (Fig. 1D). The enhanced guanylurea production in the complemented strain is likely due to the increased gene copy number and promoter activity of the introduced recombinant plasmid, which increased the expression of MetT, thereby accelerating metformin uptake and subsequently upregulating the transcription of metformin catabolic genes. However, strain NyZ550Δ*metT* exhibited no detectable production of guanylurea in the whole-cell biotransformation system (Fig. 1D; Fig. S5C). In contrast, guanylurea was indeed detected in strain NyZ550Δ*metT* when performing the degradation assay with cell lysates (Fig. 1E; Fig. S5F). Cell lysates of strain NyZ550Δ*metT* degraded metformin at a rate of 0.05 μmol h^−1^ mg protein^−1^, comparable to those of the strain NyZ550 and strain NyZ550Δ*metT*[pBB-MetT] (0.05 and 0.08 μmol h^−1^ mg protein^−1^, respectively) (Fig. 1E). Therefore, the MetT-mediated transport of metformin can be concluded as a prerequisite for its intracellular biodegradation.

### Both transporter and catabolic enzymes are essential for metformin degradation

**Fig 2:**
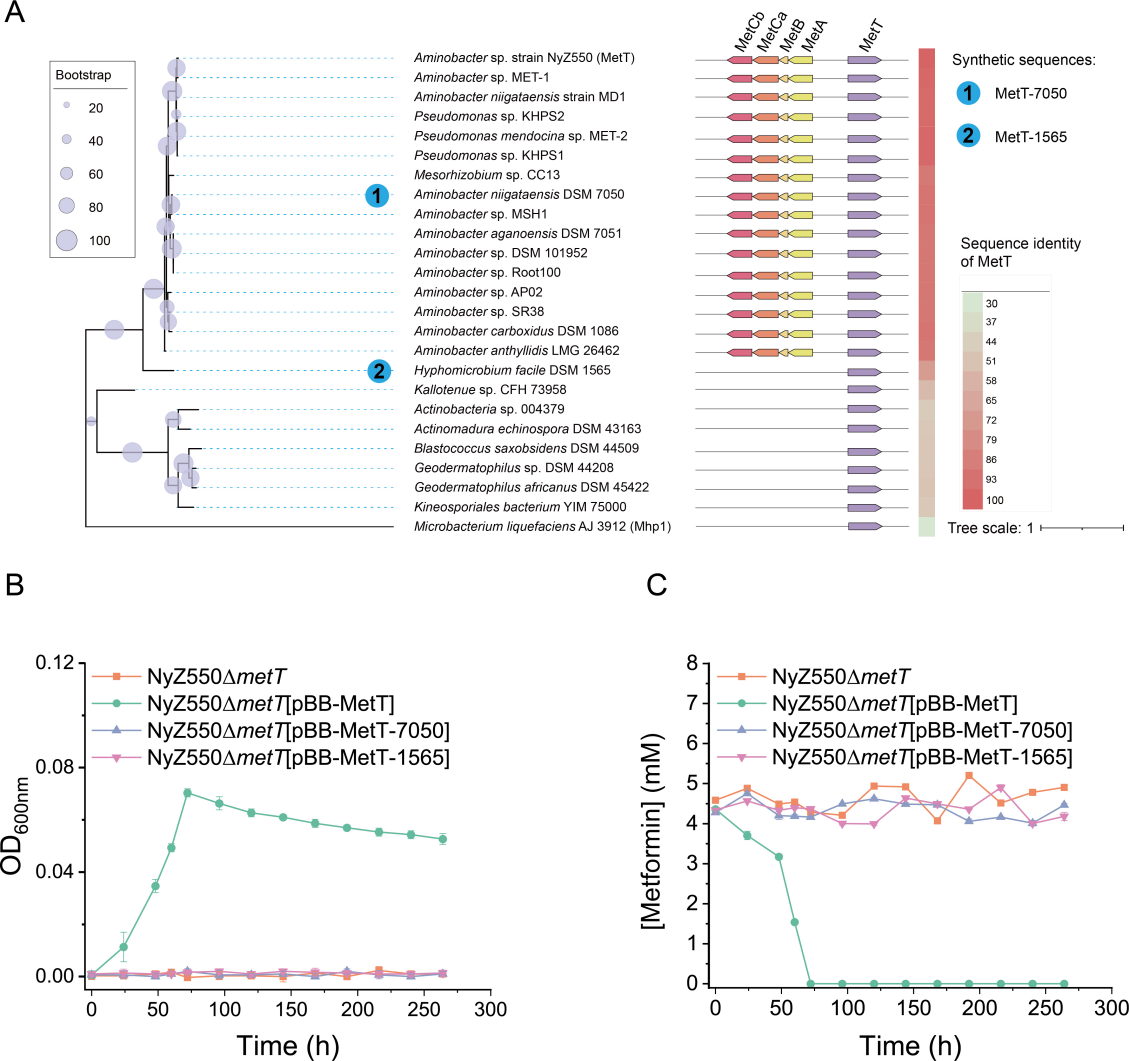
Bioinformatic and functional analyses of MetT homologs. (**A**) Phylogenetic analysis of MetT. The phylogenetic tree of MetT is shown on the left. Gene clusters encoding the transporter genes and clustered MetCaCb-like hydrolase genes are shown on the right side. The heatmap illustrates the sequence identity between MetT and its homologous proteins. Hydantoin transporter Mhp1 was used as an outgroup. Two gene arrangement patterns are displayed by Clade 1, which harbors a MetT homolog clustered with MetCaCb-like hydrolase genes, and by Clade 2, which contains the MetT homolog only. The MetT-7050 and MetT-1565 were selected from Clades 1 and 2, respectively, and their encoding genes were synthesized. (**B**) Growth curves of strain NyZ550Δ*metT* and complemented strains expressing MetT homologs cultured with metformin as the sole source of carbon, nitrogen, and energy. (**C**) Metformin degradation during growth assays to assess its utilization by the strain NyZ550Δ*metT* and complemented strains expressing MetT homologs. Values are represented as the mean ± SD from three independent experiments.

Among the genomes of currently identified metformin-degrading bacteria, encoding genes of the transporter *metT* and the catabolic enzyme *metCaCb* are always clustered. To investigate the potential relationship between *metT* and *metCaCb* among the available bacterial genomes, BLASTP searches were performed against the NCBI non-redundant (nr) protein database (36) to identify bacterial genomes that harbor homologs of both *metT* and *metCaCb*, as well as those containing *metT* alone. Remarkably, 10 strains in total were found to possess both *metT* and *metCaCb,* which have not been identified as metformin utilizers (Fig. 2A). Phylogenetic analysis of MetT proteins intriguingly revealed two distinct evolutionary paths that are correlated with the presence or absence of metformin hydrolase MetCaCb. In the genomes with MetCaCb homologs, MetT homologs are highly conserved, whereas in those without MetCaCb homologs, they display markedly lower sequence identity with MetT (Fig. 2A). This pattern indicates a functional linkage between MetT and MetCaCb in the evolution of metformin catabolism.

To assess the potential functionality of *metT* homologs from non-metformin-utilizing strains, we synthesized two putative *metT* homologous genes from *Aminobacter niigataensis* DSM 7050 (accession number: WP_183263617, with MetCaCb homolog) and *Hyphomicrobium facile* DSM 1565 (accession number: WP_092864483, without MetCaCb homolog), which were introduced into the *metT* deletion mutant strain NyZ550Δ*metT*. These two homologs are located in two separate clades, sharing 91.7% and 70.15% amino acid identity with MetT, respectively. In the growing assays, the strains NyZ550Δ*metT*[pBB-MetT-7050] and NyZ550Δ*metT*[pBB-MetT-1565] displayed no growth or metformin consumption, in contrast to the strain NyZ550Δ*metT*[pBB-MetT] (Fig. 2B and C). A previous study reported that *A. niigataensis* DSM 7050 is unable to grow on metformin, likely due to the absence of MetCaCb homologs (23). Consistently, our results demonstrated that the MetT homologs failed to restore metformin transport in strain NyZ550Δ*metT*. However, potential variations in expression level cannot currently be ruled out. Our results suggest that efficient degradation of metformin in strain NyZ550 requires both the functional transporter and downstream catabolic enzymes. These observations highlight a possible evolutionary link between substrate uptake and metabolic processing in environmental bacteria for their adaptation to synthetic compounds.

### Membrane topology and subcellular localization of MetT

The *metT* gene encodes a protein consisting of 469 amino acids, with a molecular weight of 50.29 kDa. Two widely used prediction tools, TMHMM (37) and TOPCONS (38), were employed to gain insight into the membrane topology of MetT. Both tools consistently predicted that MetT contains 12 transmembrane α-helices (Fig. S6), which is in common with the LeuT superfamily, with the N- and C-termini located on the cytoplasmic side of the membrane.

**Fig 3:**
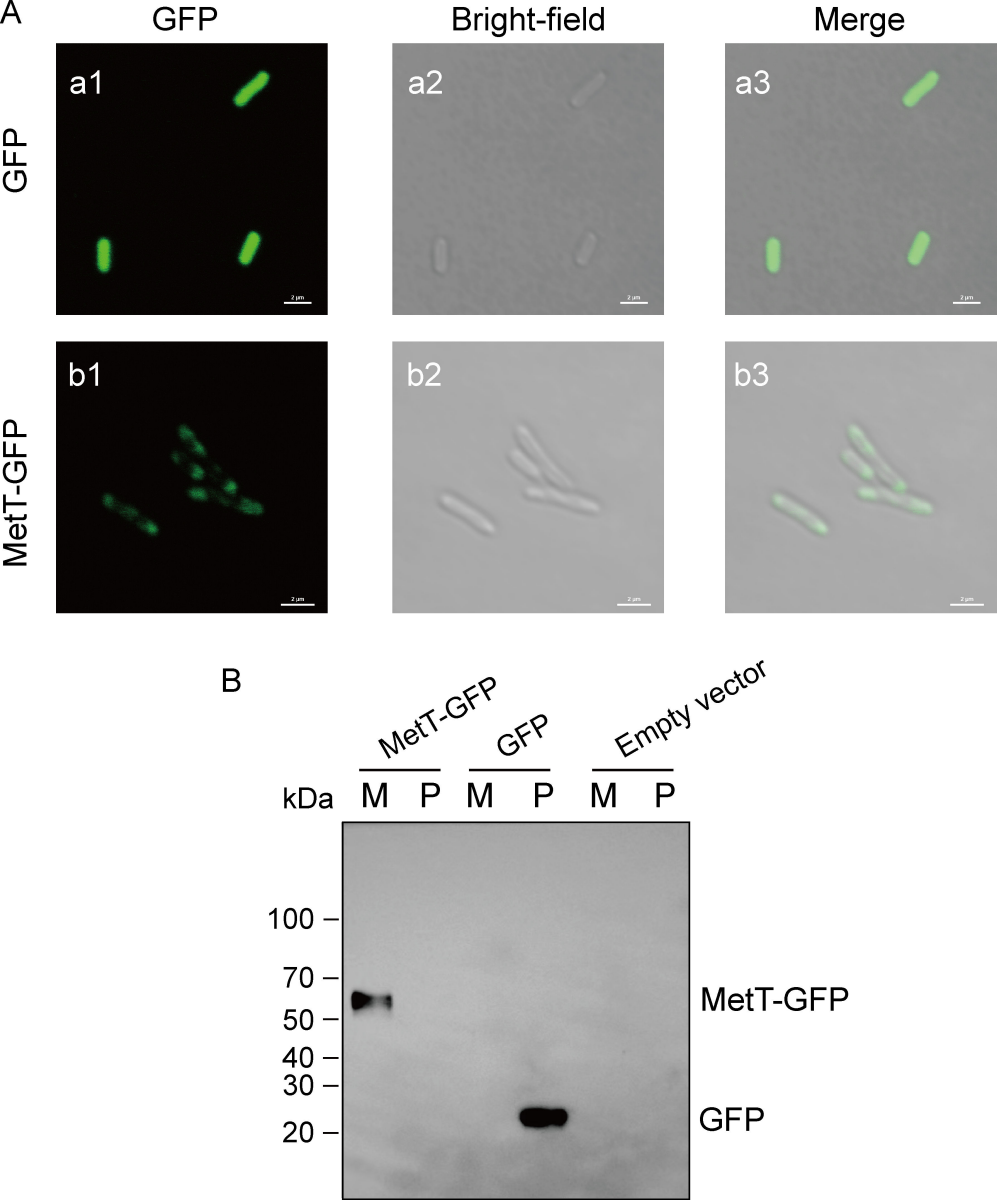
Subcellular localization of MetT. (**A**) Fluorescence microscopic localization of MetT in the *E. coli* cell envelope. The bacteria were illuminated with 488 nm light and observed at 520 nm. MetT-GFP: GFP was fused to the C-terminus of the MetT protein. GFP: GFP expressed as a non-fusion protein. (a1) Confocal images of the GFP signal in *E. coli* expressing GFP alone; (a2) bright-field image of *E. coli* expressing GFP alone; (a3) merged image shows colocalization; (b1) confocal images of GFP signal in *E. coli* expressing MetT-GFP; (b2) bright-field image of *E. coli* expressing MetT-GFP; (b3) merged field shows colocalization. (**B**) Western blot analysis to detect the subcellular localization of MetT-GFP fusion protein. M: membrane fractions; P: cytoplasmic fractions. The immunoblot shown was decorated with anti-GFP antibody.

The fusion of green fluorescent protein (GFP) to either the N- or C-terminus of membrane proteins is a commonly used strategy for monitoring their subcellular localization. To visualize the *in vivo* localization of MetT, a MetT variant with a C-terminal fused GFP (MetT-GFP) was expressed using the plasmid pTTQ18 and analyzed via fluorescence microscopy. The results revealed that the fluorescence was aggregated at the polar of the cell, rather than uniformly distributed in the whole cytoplasm, as observed for cytoplasmic GFP alone (Fig. 3A). The spatial distribution of membrane proteins in bacterial cells is a dynamic process, and their polar localization can be attributed to distinct molecular mechanisms involving either membrane lipid composition or specific protein secretion pathways (39). To further confirm the membrane localization of MetT, Western blot analysis of MetT was performed with the subcellular fractions separated from cells expressing MetT-GFP or GFP alone. MetT-GFP was observed in the membrane fraction with a molecular weight around 55.0 kDa, while GFP was predominantly in the cytoplasm (Fig. 3B). The observed molecular weight of the fused MetT-GFP protein was lower than the theoretical weight (77.06 kDa), likely due to partial unfolding of the membrane protein in SDS (40). This phenomenon has been consistently observed among various members of the NCS1 family proteins (28, 33).

### The apparent *K*_m_ of strain NyZ550 for metformin uptake

As previously mentioned, a plausible explanation for the growth and degradation deficiency of NyZ550Δ*metT* is that metformin is no longer being transported into the cells. To trace the MetT-mediated uptake of metformin by strain NyZ550 cells, we conducted an isotope tracer experiment using ^13^C^15^N-labeled metformin as the substrate. This process was measured using stable isotope ratio mass spectrometry, as indicated by intracellular isotopic carbon (^13^C) levels (δ13C_V-PDB_). The levels of δ13C_V-PDB_ were significantly increased in both strain NyZ550 and complemented strain NyZ550Δ*metT*[pBB-MetT], but not in strain NyZ550Δ*metT*, indicating that the ^13^C^15^N-labeled metformin was transported into the cells expressing MetT (Fig. S7A).

To further characterize the transport kinetics of metformin by MetT, a radioactive isotope uptake assay was performed using ^14^C-labeled metformin. Initially, the accumulation of radiolabeled metformin was measured in *E. coli* expressing MetT. However, a high background signal was observed, interfering with the measurement, which is possibly due to the presence of endogenous MetT homologs in the host *E. coli*. Alternatively, both *metT* and *metCa* genes were sequentially deleted, resulting in a mutant strain designated NyZ550Δ*metT*Δ*metCa* losing transport and catalytic activity of metformin. Subsequently, a complemented strain was constructed by reintroducing the *metT* gene into this double deletion background, generating strain NyZ550Δ*metT*Δ*metCa*[pBB-MetT], expressing recombinant MetT capable of transporting metformin, which is no longer degraded within the cell. The catabolic deficiency occurs because the functional metformin hydrolase MetCaCb necessitates the assembly of both subunits into an active heterohexameric complex; neither subunit possesses catalytic activity alone (21).

**Fig 4:**
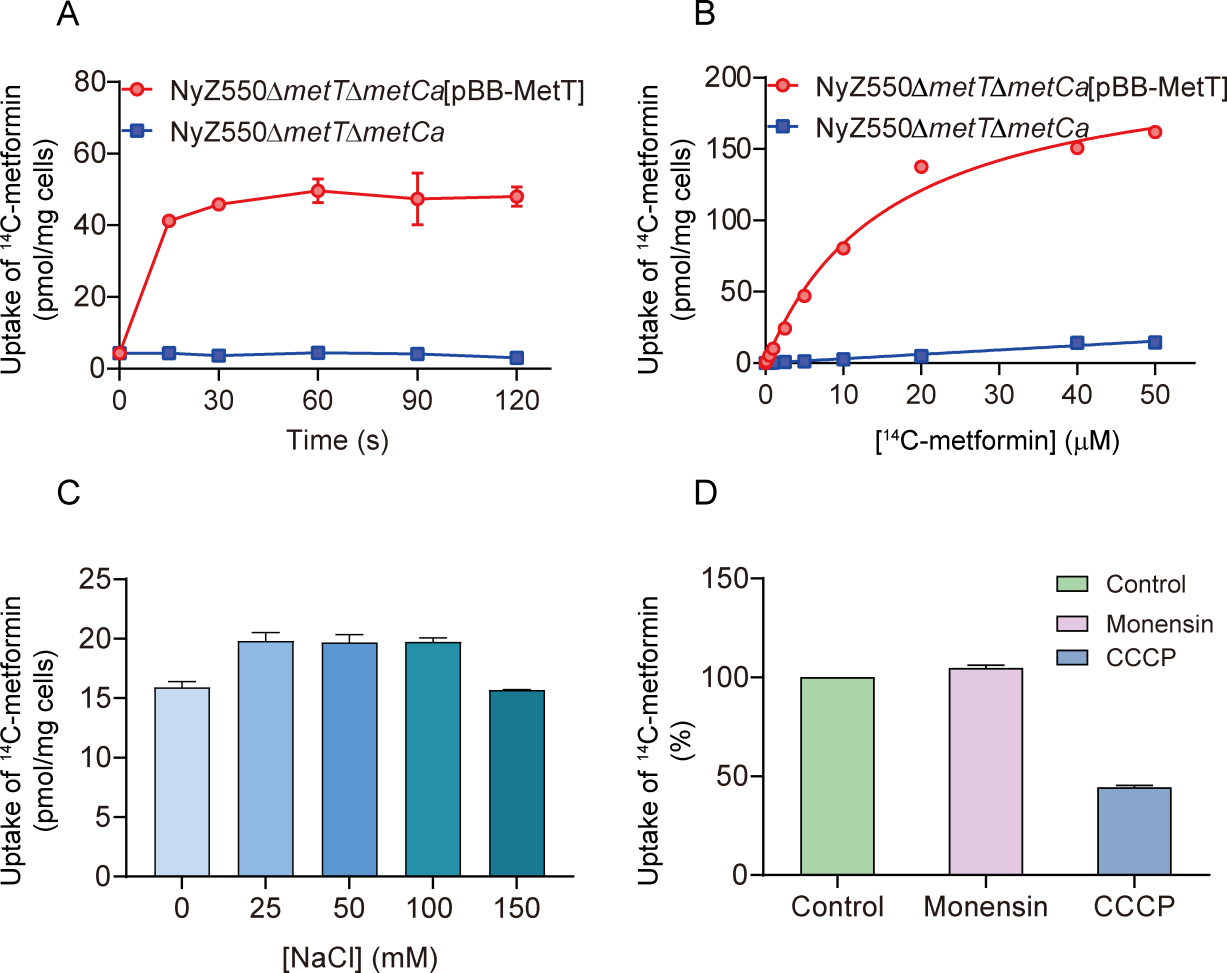
The transport properties of MetT. (**A**) Time course of ^14^C-metformin uptake in strain NyZ550Δ*metT*Δ*metCa* and the complemented strain NyZ550Δ*metT*Δ*metCa*[pBB-MetT]. (**B**) Kinetics of ^14^C-metformin transport in strain NyZ550Δ*metT*Δ*metCa* and the complemented strain NyZ550Δ*metT*Δ*metCa*[pBB-MetT]. (**C**) Uptake of ^14^C-metformin at varying sodium concentrations in the complemented strain NyZ550Δ*metT*Δ*metCa*[pBB-MetT]. (**D**) Effect of sodium ionophore monensin and the protonophore CCCP on metformin uptake by MetT. The control group, representing uninhibited MetT transport activity, was set to 100% (16.08 ± 0.43 pmol/mg cells) for normalization. Values are presented as the mean ± SD of two independent experiments.

The cellular uptake of ^14^C-labeled metformin was quantitatively analyzed in strain NyZ550Δ*metT*Δ*metCa*[pBB-MetT] and its negative control, NyZ550Δ*metT*Δ*metCa*. Time-course analysis revealed that cells expressing MetT (NyZ550Δ*metT*Δ*metCa*[pBB-MetT]) were able to accumulate ^14^C-labeled metformin (Fig. 4A), and Michaelis-Menten kinetic analysis revealed an apparent affinity for substrate transport (*K*_m_) of 15.90 ± 1.75 µM and a maximum transport velocity (*V*_max_) of 217.3 ± 9.21 pmol/mg cells (Fig. 4B). No transport activity was detected from the strain lacking *metT* (NyZ550Δ*metT*Δ*metCa*) (Fig. 4A).

Members of the NCS1 family function as either proton-coupled or sodium-coupled substrate symporters, mediating the transport of specific substrates across biological membranes (26). Therefore, the effects of the sodium ion and proton gradient on metformin uptake were preliminarily tested here. Under conditions of varying sodium ion concentrations, the uptake rate of ^14^C-labeled metformin remained constant across the entire range of tested sodium chloride concentrations from 0 to 150 mM (Fig. 4C). Furthermore, the sodium ionophore monensin exhibited no impact on metformin transport (Fig. 4D). Due to the technical challenge of establishing a proton gradient across an intact cellular membrane, the effect on metformin uptake was tested by disrupting the proton gradient using the protonophore carbonyl cyanide *m*-chlorophenylhydrazone (CCCP). It was found that the CCCP significantly inhibited metformin uptake by strain NyZ550Δ*metT*Δ*metCa*[pBB-MetT] (Fig. 4D). These findings suggest that MetT is likely a proton-coupled symporter, utilizing proton motive force rather than sodium gradient for substrate translocation.

### Phylogenetic relationship of MetT and its homologs

**Fig 5:**
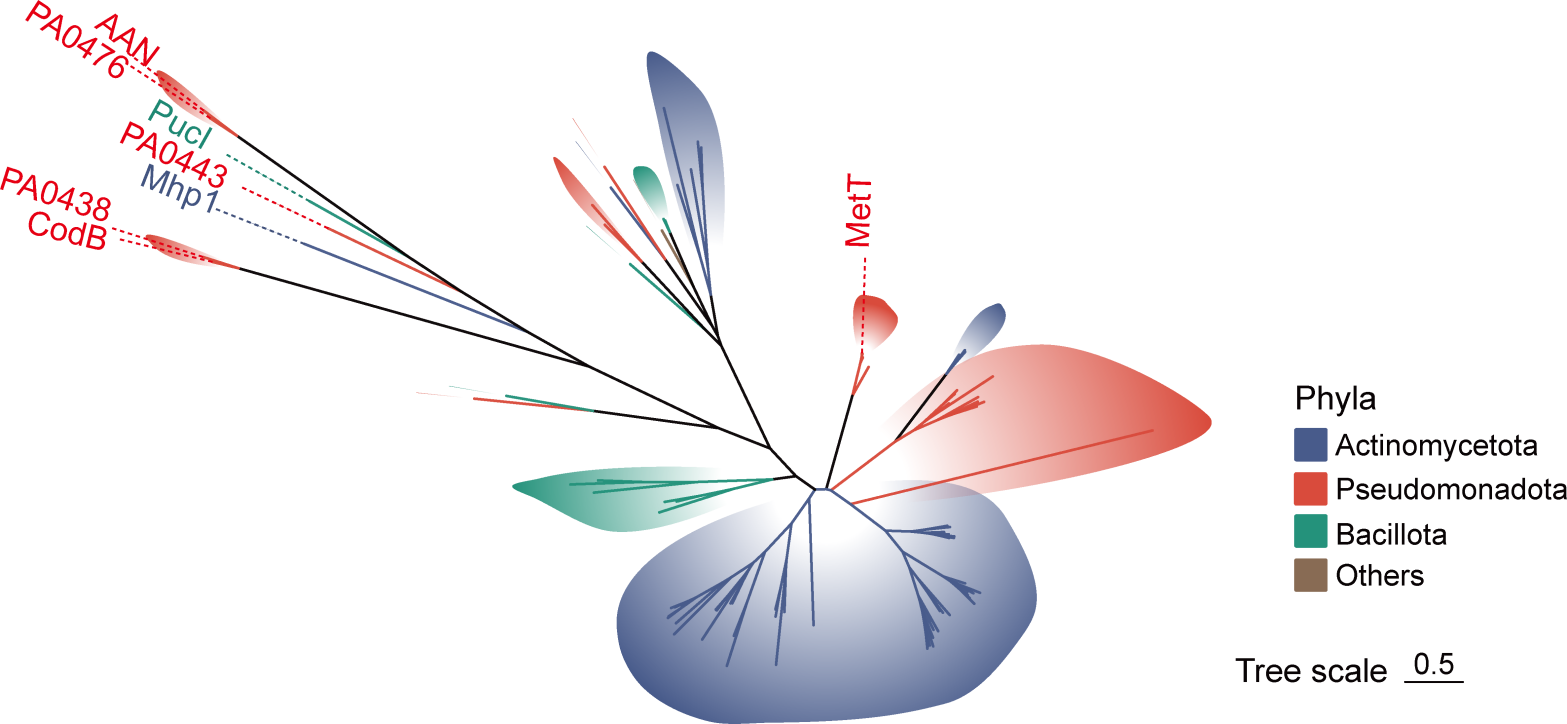
The phylogenetic tree of MetT along with other experimentally characterized bacterial NCS1 family transport proteins. The well-studied NCS1 proteins are Mhp1 (UniProt: D6R8X8), PucI (UniProt: P94575), CodB (UniProt: P0AA82), PA0438 (UniProt: Q9I679), PA0476 (UniProt: Q9I642), PA0443 (UniProt: Q9I674), and AAN (UniProt: Q88EZ1). The color of each protein label corresponds to the phylum to which it belongs. The phylogenetic tree explicitly displays phyla with an abundance above 5%, while the remainder are categorized as “others”.

BLASTp analyses were conducted using MetT as the query sequence (with an *E*-value <1e-5 and sequence identity >30%) to identify and evaluate the conservation of MetT homologs across microbial genomes. Our analysis uncovered MetT homologs encoded by various bacterial phyla, including *Actinobacteriota*, *Pseudomonadota,* and *Bacillota*. To elucidate the phylogenetic position of MetT within the NCS1 family, a maximum-likelihood phylogenetic tree was constructed using MetT homologs and functionally characterized NCS1 transporters. These selected reference transporters are known to facilitate the uptake of nucleobases and related compounds, including Mhp1 (hydantoin transporter) (28), CodB (cytosine transporter) (32), PuCI (allantoin transporter) (33), AAN (allantoin transporter) (41), PA0438 (cytosine and 5-fluorocytosine transporter), PA0476 (allantoin transporter), and PA0443 (cytosine, thymine, uracil, and dihydrouracil transporter) (35). Phylogenetic reconstruction (Fig. 5) and sequence similarity network (SSN) analysis (Fig. S8) both demonstrated that MetT is an evolutionarily distinct lineage within the NCS1 family, exhibiting no association with any of the known characterized functional clusters. It implies that MetT forms a distinct NCS1 subfamily with novel substrate specificity. The other experimentally characterized bacterial NCS1 proteins exhibit substrate specificity primarily toward pyrimidine and hydantoin derivatives, which share the common feature of containing nitrogenous heterocyclic rings. Given the fact that metformin is structurally defined as a straight-chain biguanide derivative that does not contain any heterocyclic rings, it is likely that MetT has evolved from typical NCS1 family proteins to accommodate a distinct substrate, metformin, while maintaining its overall conserved structural framework.

### Transportation substrates of MetT

In the phylogenetic analysis, all detected homologs of MetT were classified within the NCS1 family. NCS1 transporters are generally known to facilitate the salvage of nucleobases from the environment, thereby conserving energy by circumventing *de novo* nucleotide synthesis (26). Disruption of these transporters could compromise microbial assimilation and utilization of these compounds (35). To investigate whether MetT is also implicated in the uptake of nucleobase analogs, a growth-based substrate utilization assay was conducted to compare the growth pattern of strain NyZ550 with that of its *metT* knockout derivative on various nucleobase analogs. It turned out that the deletion mutant displayed similar growth characteristics to those of the wild type (Fig. S9), suggesting either an incapacity of MetT to transport nucleobase analogs or the presence of functional redundancy among nucleobase transporters in strain NyZ550.

**Fig 6:**
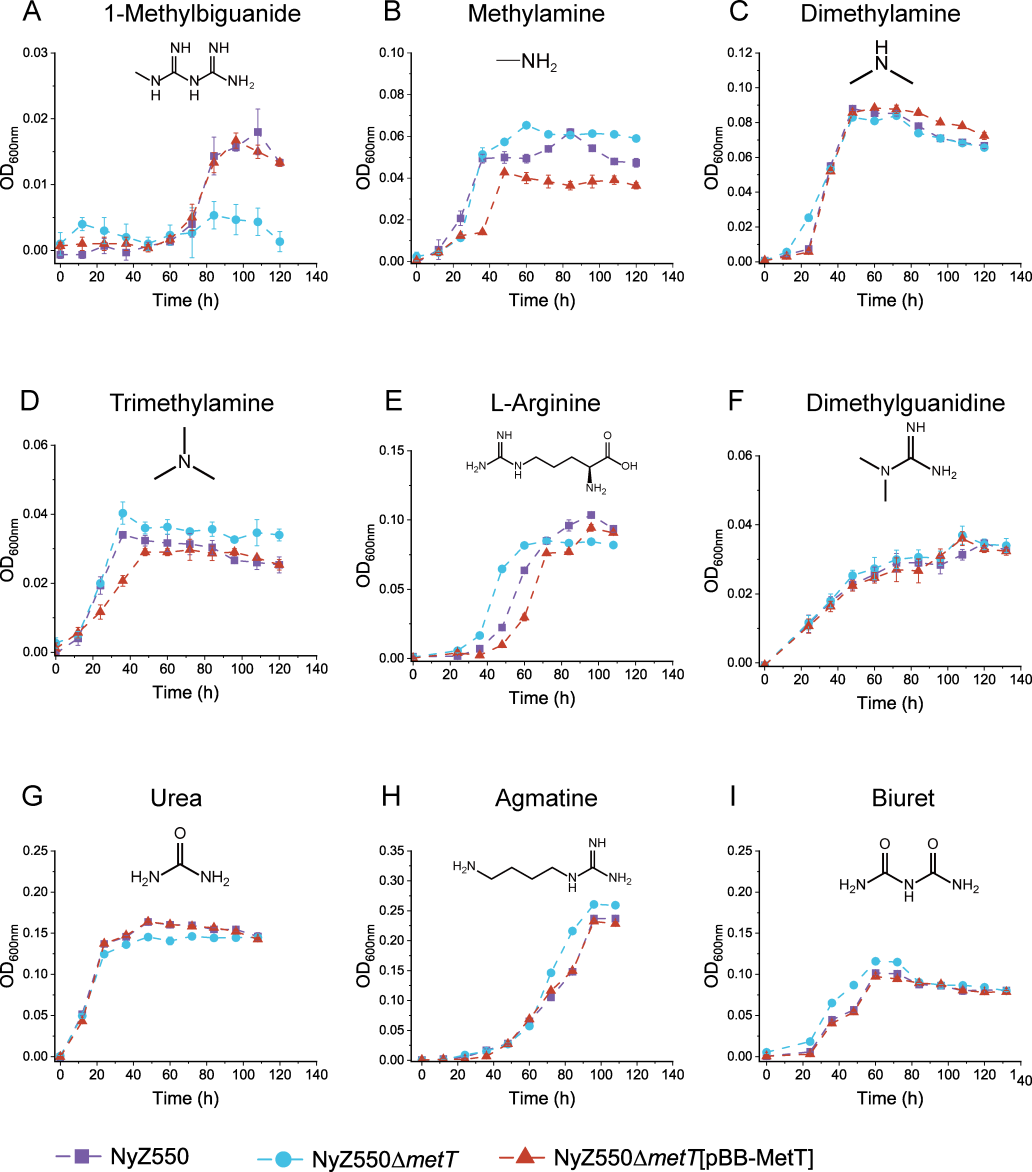
Growth of strain NyZ550, strain NyZ550Δ*metT*, and the complemented strain NyZ550Δ*metT*[pBB-MetT] on a variety of nitrogenous compounds to reveal whether MetT is essential for its growth. (**A–E**) Compounds (4 mM each) indicated were used as the sole source of carbon and nitrogen. (**F–I**) Compounds (2 mM each) indicated were used as the sole source of nitrogen, supplemented with 10 mM glucose as the carbon source. Among them, MetT is required for strain NyZ550 to grow only when using 1-methylbiguanide. Data are presented as the average mean ± SD from *n* = 3 independent experiments.

Considering the ability of strain NyZ550 to utilize a wide array of nitrogenous compounds as growth substrates (42), we subsequently investigated whether MetT might be involved in the transport of additional nitrogen-containing molecules. Growth assays indicated the mutant strain NyZ550Δ*metT* was able to grow on all selected compounds that the WT strain could utilize (Fig. 6B through I), except for 1-methylbiguanide (Fig. 6A), a structural analog of metformin differing only by the absence of a single methyl group at the N1-position. This growth defect was restored through genetic complementation using the *metT* gene, confirming that MetT is necessary for the uptake of 1-methylbiguanide in strain NyZ550, apart from metformin. Collectively, these findings firmly establish that the uptake and metabolism of metformin and 1-methylbiguanide in strain NyZ550 are strictly dependent on MetT.

### Structural insights into substrate transport by MetT

**Fig 7:**
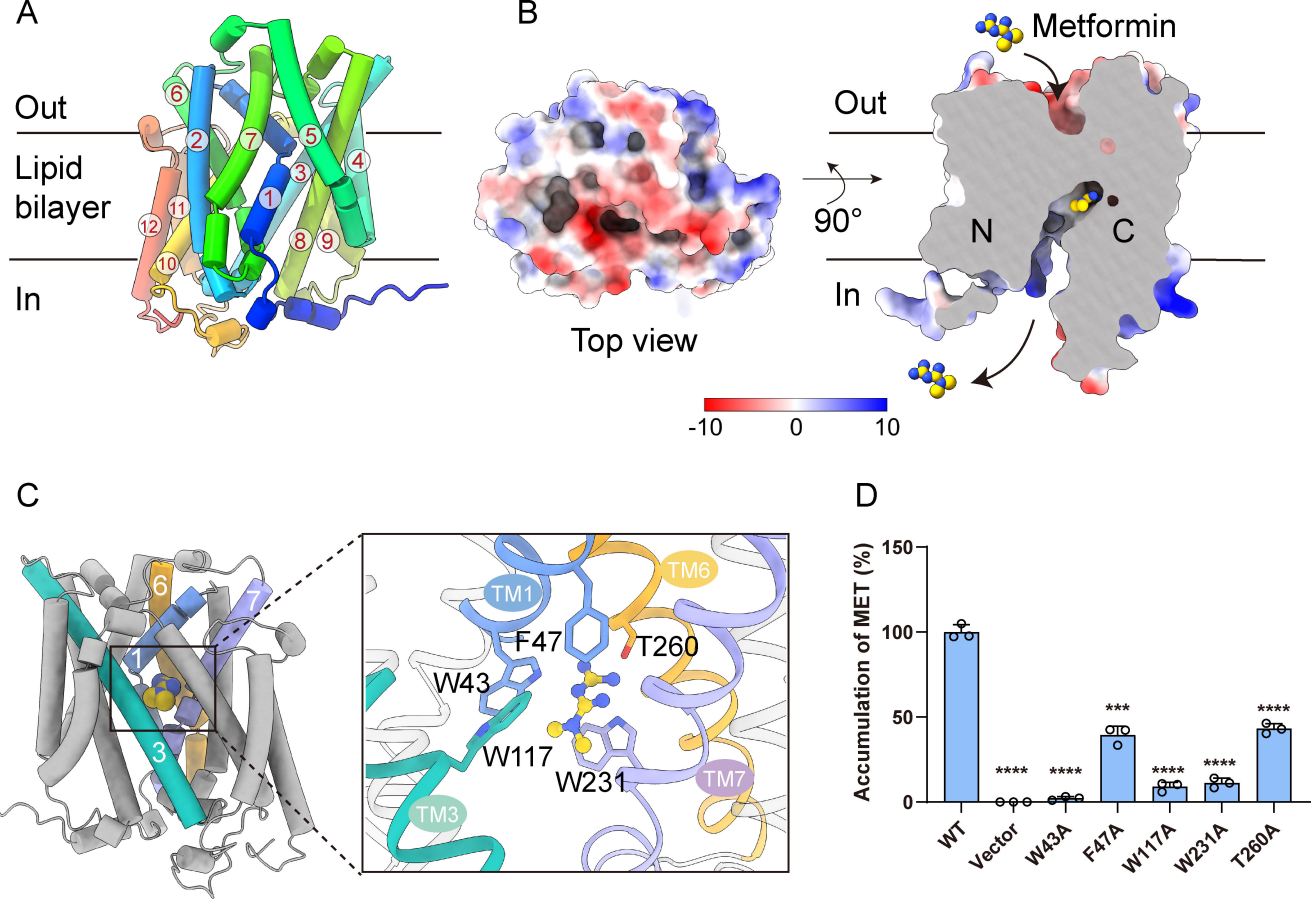
The proposed binding mode of MetT with metformin. (**A**) The overall structure of MetT was predicted by Alphafold3. The structure of MetT is viewed in the plane of the membrane. The helices on the inside and outside of the membrane are indicated as “in” and “out”, respectively. (**B**) The electrostatic potential of MetT. The surface of the MetT protein is colored according to the electrostatic potential calculated by ChimeraX. Surface coloring is indicated in the scale bar. The top view of MetT shows a strong negative charge. The arrow indicates the direction of metformin transport from the extracellular space, across the MetT transporter, into the intracellular space. (**C**) The binding mode of metformin within MetT. Residues W43, F47, W117, W231, and T260 were found to be crucial for the substrate binding. (**D**) Initial uptake rates of metformin by MetT mutants. Uptake in wild-type MetT is set as 100% (20.14 ± 0.89 pmol/mg cells). Values are presented as the mean ± SD of three independent experiments. Statistical significance between mutants and wild-type MetT was determined using Student’s t-test and is denoted as ***P  <  0.001, ****P  <  0.0001.

To investigate the substrate transport mechanism of MetT, we combined protein structure prediction (43) with molecular docking (44, 45) of metformin into the predicted model. Predicted MetT structure exhibits the canonical LeuT superfamily topology (46), with 12 transmembrane helices (TMs) adopting an inward-open state (Fig. 7A; Fig. S10). Electrostatic potential distribution analysis revealed a negative electrostatic potential field in the extracellular surface, which likely mediates the initial recognition and orientation of the protonated metformin through complementary charge interactions (Fig. 7B). Following its initial recognition, molecular docking suggests that metformin moves into the central cavity, which is composed of transmembrane helices TM1, TM3, TM6, and TM7 (Fig. 7C; Fig. S12). Notably, the central cavity constitutes a hydrophobic pocket enriched in aromatic residues, including W43, F47, W117, W231, and T260, likely stabilizing metformin via cation–π interactions (Fig. 7C). To assess the functional significance of these residues, site-directed mutagenesis was performed by introducing alanine substitutions into the double knockout strain NyZ550Δ*metT*Δ*metCa*. Intracellular metformin concentrations in the mutant strains were quantitatively determined by ultra-performance liquid chromatography mass spectrometry (UPLC-TOF-MS). All MetT mutants showed reduced metformin transport efficiency, with W43A, W117A, and W231A retaining only 2.2%, 9.0%, and 11.2% of original activity, respectively (Fig. 7D). Furthermore, a tunnel between the N-terminal domain (NTD) and C-terminal domain (CTD) is clearly visible, suggesting a pathway for metformin to release into the cell (Fig. 7B). Of note, a similar approach of metformin transport is also adopted in the human metformin transporter OCT1, suggesting a conserved strategy for cationic metformin transport (47).

## DISCUSSION

It is generally acknowledged that environmental microorganisms evolve specialized enzymatic systems to degrade various synthetic compounds. For many compounds, especially those with low membrane permeability, the presence of dedicated transporters is essential to facilitate substrates’ entry into the cell, serving as a critical prerequisite for efficient biodegradation (48). For instance, 3-(3-hydroxyphenyl)propionate (3HPP) transporter MhpT has been shown to be indispensable for the uptake and subsequent degradation of 3HPP in *E. coli* K-12 (49). Similarly, the degradation of hydrophobic pollutants like petroleum hydrocarbons relies on specialized outer membrane transport systems, including TonB-dependent transporters (5), OmpW family proteins (50), and FadL family channel proteins (51). Here, the critical role of membrane transporters is also exemplified in the biodegradation of metformin. In this study, we characterized MetT, a member of the NCS1 family transporter in *Aminobacter* sp. strain NyZ550, as a specific metformin transporter. Unlike conventional NCS1 family transporters, which typically recognize purine or pyrimidine analogs as substrates, MetT transports a structurally distinct guanidine derivative metformin, with a high substrate affinity.

Phylogenetic and SSN analyses positioned MetT in an evolutionarily distinct lineage within NCS1 transporters, highlighting a significant evolutionary innovation in microbial transporter systems (Fig. 5). NCS1 transporters play a fundamental role in microbial metabolism by mediating nucleobase salvage, thereby reducing the energetic cost of *de novo* nucleotide synthesis (52). These transporters are typically associated with the uptake of naturally occurring nucleobases, particularly purine and pyrimidine analogs containing nitrogenous heterocycles (26). In contrast, MetT mediates the transport of metformin, a synthetic linear biguanide derivative lacking heterocyclic rings, representing a distinct substrate category for the NCS1 family. This finding significantly expands the scope of functional NCS1 transporters and demonstrates that the conserved structural scaffold of this family can evolve to accommodate emerging anthropogenic compounds, such as metformin.

The NCS1 family is driven by certain energy-coupling mechanisms, primarily involving H^+^ or sodium ion Na^+^ gradients. The hydantoin transporter Mhp1 and the cytosine transporter CodB utilize Na^+^ gradients for substrate translocation, whereas the allantoin transporters PucI and AAN rely on H^+^ gradients (29, 32, 33, 41). In this study, the uptake of ¹⁴C-metformin by MetT was inhibited by the proton uncoupler CCCP, a characteristic indicative of proton-dependent transporters (53, 54), although the underlying mechanism of proton interaction remains unresolved. Structural modeling and docking analyses in this study revealed that MetT and the structurally verified hydantoin transporter Mhp1 share a similar overall transmembrane framework for substrate transport, with TM1, TM3, and TM6 contributing to substrate recognition (Fig. S11). However, the specific residues of MetT and Mhp1 mediating substrate interactions differ, reflecting adaptation to their distinct substrates (Fig. S11). In Mhp1, the benzyl-hydantoin binding pocket is delineated by transmembrane helices TM1, TM3, TM6, and TM8 (Fig. S11) (29). This pocket is formed by key residues, specifically A38, W117, Q121, W220, N314, and N318 (Fig. S11) (29). By contrast, docking of metformin to MetT indicated TM1, TM3, TM6, and TM7 as key contributors (Fig. S11). Furthermore, the binding cavity was found to be surrounded by aromatic residues W43, F47, W117, W231, and T260 (Fig. 7C). While variations in expression levels cannot be ruled out, the marked reduction in metformin uptake efficiency following site-directed mutagenesis underscores the pivotal involvement of these residues in the transport process (Fig. 7D). This functional significance of these residues is further corroborated by the structure and sequence analysis of non-functional homologs, MetT-7050 and MetT-1565. Despite sharing a conserved structural scaffold (RMSD of 0.394 Å and 0.508 Å, respectively), these homologs exhibit F47L and T260A substitutions at pivotal substrate-binding positions identified in MetT (Fig. S13). The absence of metformin transport activity in these homologs is likely attributable to these naturally occurring variations in key binding interactions. Collectively, these findings suggest that while the structural scaffold of substrate recognition is conserved, the positioning of interacting residues has diverged to accommodate the distinct physicochemical properties of benzyl-hydantoin and metformin.

In human beings, the plasma concentration of metformin ranges from 5.16 to 9.04 mg/L (approximately 40–70 μM) (11). Metformin transporters in humans display similar affinity profiles for metformin, with respective *K*_m_ values of 1.44 mM (OCT1), 1.07 mM (OCT2), 1.10 mM (OCT3), 1.32 mM (PMAT), 0.23 mM (MATE1), and 1.05 mM (MATE2-K) (55). These values indicate a relatively low affinity for metformin, which is consistent with the fact that these transporters are non-selective carriers for a range of organic cations, zwitterions, and even some uncharged compounds (56). In contrast, bacterial-originated metformin transporter MetT in this study exhibited a significantly higher affinity for metformin, with a *K*_m_ value of 15.90 ± 1.75 µM (Fig. 4B). To survive in oligotrophic environments, microorganisms appeared to have evolved highly specific transporters that enhance the efficiency of nutrient acquisition. For example, marine SAR11 bacteria have ultra-high-affinity solute-binding proteins that allow for efficient assimilation of dissolved organic matter, meeting their needs for carbon, nitrogen, sulfur, and phosphorus requirements (57). The concentration of metformin in environments varies widely, ranging from 0.8 nM to 3.8 μM in the wastewater of WWTPs in our previous study (42). The evolutionary development of high-affinity transport systems allows bacterial cells to be able to capture and internalize this scarce substrate at micromolar concentrations, providing a crucial survival advantage in nutrient-limited environments. The marked difference in affinity between MetT and human transporters highlights a potential divergence in substrate recognition and transport mechanisms.

In summary, we characterized a highly specific bacterial metformin transport system, highlighting the central role of microbial membrane transporters in drug metabolism. Our study demonstrates that the efficient biodegradation of metformin depends not only on catabolic enzymes but also on a specialized transporter. Through structural modeling and site-directed mutagenesis, we identified key residues critical for substrate recognition. However, the absence of an experimentally resolved MetT structure currently limits a full mechanistic understanding of its transport dynamics. Future studies will be crucial to elucidate the structural and functional adaptations of this metformin transporter, which govern the uptake and catabolism of metformin at trace environmental concentrations.

## MATERIALS AND METHODS

### Chemicals, bacterial strains, and culture conditions

[Biguanidine-^14^C]metformin hydrochloride (100 mCi/mmol) was purchased from Pharmaron (Beijing, China). Metformin (1,1-dimethylbiguanide) and guanylurea were obtained from TCI (Shanghai, China). Biuret and 1-methylbiguanide were purchased from Bidepharm (Shanghai, China). Dimethylguanidine was obtained from YuanYe Bio-Technology (Shanghai, China). 1-Methylamine, dimethylamine (40% in water), trimethylamine (30% in water), and CCCP were purchased from Sigma-Aldrich (St. Louis, USA). L-arginine was purchased from BBI CO. (Shanghai, China). Monensin was purchased from Sinopharm (Shanghai, China). The anti-GFP antibody (Cat# SLAB3001) was purchased from Smart-Lifesciences (Changzhou, China), and the secondary antibody anti-rabbit-HRP (Cat# ZB-2301) was obtained from ZSGB-Bio (Beijing, China). The bacterial strains and plasmids used in this study are listed in Table 1. *Aminobacter* sp. strain NyZ550 was isolated as previously described (42). Strain NyZ550 was routinely cultured in 1/5 strength tryptic soy broth (TSB) at 30°C in a rotary shaker. When assessing the substrate utilization capacity, strain NyZ550 and its derivatives were cultured at 30°C in minimal salt medium (MSM, pH 7.0) containing metformin (2–10 mM) as the sole source of carbon, nitrogen, and energy. *Escherichia coli* strains were grown in lysogeny broth (LB) medium at 37°C with shaking at 180 rpm. Ampicillin and gentamicin were used, when necessary, at final concentrations of 100 and 10 μg/mL, respectively.

**TABLE 1:** Bacterial strains, plasmids, and primers

Material	Description or sequence (5′−3′)	Source or reference
Bacterial strains		
*E. coli* C43 (DE3)		Adamas-Life
Strain NyZ550	Wild-type; metformin-degrading strain	(42)
NyZ550Δ*metT*	Strain NyZ550 with *metT* deletion	This study
NyZ550Δ*metT*Δ*metCa*	Strain NyZ550 with *metT* and *metCa* deletion	This study
NyZ550Δ*metT*[pBB-MetT]	Strain NyZ550Δ*metT* complemented with *metT*	This study
NyZ550Δ*metT*Δ*metCa*[pBB-MetT]	Strain NyZ550Δ*metT*Δ*metCa* complemented with *metT*	This study
Plasmids		
pEX18Gm	Gm^R^; oriT^+^sacB^+^, gene replacement vector with MCS from pUC18	(58)
pBBR1MCS-2	Gm^r^, pBBR1 replicon, mob^+^	(59)
pTTQ18	IPTG inducible co-expression vector, Amp^r^	(60)
pEX-HA-MetT	The knockout vector of the upstream and downstream homologous arms (HA) of metT	This study
pBB-MetT	pBBR1MCS-2 derivative for *metT* expression; Gm^r^	This study
pTTQ18MetT-GFP	pTTQ18 derivative for the expression of *metT* fused with GFP; Amp^r^	This study
pTTQ18-GFP	pTTQ18 derivative for the expression of GFP; Amp^r^	This study
Primers		
MetT-F	GAATCTGGCTGCCGGTTTTG	Forward primer (FP) for RT-qPCR of *metT*
MetT-R	TTCGCCGAGAACAGTAGCAG	Reverse primer (RP) for RT-qPCR of *metT*
16S-F	GAAATCCCAGGGCTCAAC	FP for RT-qPCR of reference genes
16S-R	CGCACCTCAGCGTCAGTA	RP for RT-qPCR of reference genes
LHA_MetT-F	TATGACCATGATTACGAATTCAAAACAGGACGATGCCAACCC	FP for left homology arm (LHA) of *metT*
LHA_MetT-R	GTCGTTGAAGAACAGCGTGACCCCGAGG	RP for LHA of *metT*
RHA_MetT-F	TCACGCTGTTCTTCAACGACGGTGCCGA	FP for right homology arm (RHA) of *metT*
RHA_MetT-R	ACGACGGCCAGTGCCAAGCTTTTGCAGCAGGTAGAGGAAGACG	RP for RHA of *metT*
LHA_MetCa-F	CCATGATTACGAATTCGCGTTCTGTTCATCGAAAATGTCG	FP for LHA of *metCa*
LHA_MetCa-R	CTCCTCCCTGAGAATTCGCTGTTCCTCCCAA	RP for LHA of *metCa*
RHA_MetCa-F	TGGGAGGAACAGCGAATTCTCAGGGAGGAGATTAAGTCATG	FP for RHA of *metCa*
RHA_MetCa-R	GGCCAGTGCCAAGCTTCGTTGAAATTGAAGCCGAGGTACT	RP for RHA of *metCa*
pBB-MetT-F	GATAAGCTTGATATCGAATTCATGGCGTCCCAACCCAAT	FP for *metT* expression
pBB-MetT-R	CGCTCTAGAACTAGTGGATCCTCAATATCCTCCGCGCCG	RP for *metT* expression
pTTQ-MetT-GFP-F	GAAACAGCGATGAATTCAATGGCGTCCCAACCCAAT	FP for *metT-gfp* expression
pTTQ-MetT-GFP-R	CCAAGCTTGCATGCCTGCAGTCATTTGTAGAGCTCATCCATGCCATGTGTAA	RP for *metT-gfp* expression
pTTQ-GFP-F	AGGAAACAGCGATGAATTCATGAGCAAAGGAGAAG	FP for *gfp* expression
pTTQ-GFP-R	CCAAGCTTGCATGCCTGCAGTCATTTGTAGAGCTCATCCATGCCA	RP for *gfp* expression

### Generation of gene knockout and complemented strains

Gene knockout of strain NyZ550 was constructed using a two-step homologous recombination method. For *metT* deletion, the upstream and downstream of the target gene were amplified and fused together by overlap extension PCR. The primers used are listed in Table 1. The resulting fragment was ligated into the *Eco*RI- and *Bam*HI-digested plasmid pEX18Gm, a derivative of the suicide vector pEX18Tc in which the tetracycline resistance gene cassette had been replaced with a gentamicin resistance gene cassette. The resultant pEX18Gm derivative (pEX-HA-MetT) was introduced into the diaminopimelic acid (DAP) auxotrophic *E. coli* strain WM3064, which was used as a donor for transferring the plasmid pEX-HA-MetT into strain NyZ550 by conjugation. The first-round double-crossover recombinant transconjugants containing the suicide plasmids were subsequently selected on 1/5 TSB agar plates supplemented with 10 μg/mL gentamicin, followed by counter-selection on 1/5 TSB agar containing 15% sucrose for the second recombination. Both first- and second-round recombinant strains were verified by PCR and Sanger sequencing. The double-knockout mutants were constructed by sequential gene deletion using a homologous recombination strategy similar to that described above. Regarding gene complementation, the genes (*metT* or its mutants) were cloned by PCR and ligated to *Eco*RI- and *Bam*HI-digested plasmid pBBR1MCS-2. The recombinant plasmids were introduced into the respective knockout strains using the conjugation method described above.

### RNA extraction and RT-qPCR

Total RNA was extracted from strain NyZ550 at 24, 36, and 48 h under metformin-treated and untreated conditions using the MiniBEST Universal RNA Extraction Kit (Takara, China). To eliminate genomic DNA contamination, the extracted RNA was treated with a gDNA Eraser (Yeasen, China) at 42°C for 2 min. First-strand cDNA was synthesized from 1 μg of total RNA using Hifair III Reverse Transcriptase (Yeasen, China) in accordance with the manufacturer’s protocol. RT-qPCR was performed on a CFX96 Real-Time PCR Detection System (Bio-Rad, USA) using TB Green Premix Ex Taq II (Tli RNaseH Plus) (Takara, China), with the synthesized cDNA as the template. The primers used are shown in Table 1. All reactions were conducted in triplicate. The 16S rRNA gene of strain NyZ550 was used as the internal control, and the relative expression levels of target genes were calculated using the 2^−ΔΔCt^ method (61).

### Metformin hydrolase activity assays and analytical methods

Strain NyZ550 and its derivatives were grown on TSB and harvested as described above. The pellets were resuspended in 20 mM Tris-HCl buffer (pH 8.0) to an optical density (OD_600 nm_) of 0.8. Each sample was split equally into two parts. One part was used for the resting cell biotransformation assay, and the other part was used for preparing the cell extract after ultrasonication. Subsequently, both the resting cells and the cell extract were incubated with 1 mM metformin at 30°. Samples were collected at appropriate intervals, and the hydrolytic product guanylurea was measured by UPLC-TOF-MS. The protein concentrations were quantified by the BCA protein assay kit (Beyotime, China).

Metformin and guanylurea were quantified using ultra-performance liquid chromatography (UPLC, Agilent 1290 Infinity II, USA) coupled with a time-of-flight mass spectrometer (TOF-MS) equipped with an electrospray ionization (ESI) source. Chromatographic separation was achieved on a Poroshell120 120 HILIC-Z column (2.7 μm × 100 mm × 2.1 mm). Mobile phase A consisted of ultrapure water containing 10 mM ammonium formate (adjusted to pH 3.5 with formic acid), and mobile phase B was 100% acetonitrile. For the UPLC method, the gradient elution was performed as follows: initial conditions were 50% B for 4 min, followed by a linear increase to 95% B at 8 min, which was held until 9 min. The mobile phase was then adjusted to 5% B at 10 min and equilibrated for an additional 4 min. The flow rate was 0.2 mL/min, and the injected volume was 10 μL. TOF-MS was performed in negative ion mode with a mass range of 50–500 *m/z*.

### Analysis of MetT cellular localization by confocal microscope

The full-length *metT* gene was amplified from the genomic DNA of strain NyZ550, and the *gfp* gene was amplified from the genomic DNA of *E. coli* K12. Then, a *metT-gfp* fused-DNA fragment, encoding MetT with a C-terminal fused GFP, was generated by PCR. The fusing fragment was ligated into *EcoR*I- and *Pst*I-digested plasmid pTTQ18 and transformed into *E. coli* C43 (DE3). The cells were grown at 37°C, 220 rpm in LB media supplemented with ampicillin. When the OD_600 nm_ value reached 0.6, 0.4 mM IPTG was added to induce protein expression for another 4 h of incubation at 30°C. The cells were harvested and washed three times with phosphate-buffered saline (PBS; pH 7.4). Subsequently, the cells were immobilized on microscope slides pre-coated with 1% agarose, and GFP signal was visualized using a Nikon confocal microscope (Nikon Ni-E A1 HD25).

### Cell fractionation and Western blot analysis

Cell fractionation was performed according to a previously established protocol with minor modifications to optimize the separation of cellular components (62). *E. coli* C43 (DE3) strains containing empty vector pTTQ18, vector expressing GFP, or GFP-MetT were grown and harvested as described above. The cell pellets were suspended in 0.5 mL of periplasting buffer (20% sucrose, 1 mM EDTA, 30,000 U/mL Ready-Lyse lysozyme). The cell suspensions were incubated on ice for 5 min to facilitate cell wall digestion. Spheroplasts were subsequently pelleted by centrifugation at 12,000 × *g* for 2 min, and the supernatant was collected as the periplasmic fraction. The pelleted spheroplasts were lysed in 1 mL of PBS buffer supplemented with 400 U/mL Omnicleave endonuclease. The samples were incubated at room temperature for 5 min and then subjected to sonication at 30%–40% amplitude for 8 min. Unlysed cells and debris were removed by centrifugation at 12,000 × *g* for 10 min. The resulting supernatant was further centrifuged at 100,000 × *g* for 3 h to separate the cytoplasmic and membrane fractions. The final supernatant was collected as the cytoplasmic fraction, while the precipitate was resuspended in an equal volume of PBS buffer containing 1% DDM to obtain the membrane fraction.

The Western blot analysis was performed to detect GFP in each fraction. Specifically, cell fractions were analyzed by SDS-PAGE and transferred onto a polyvinylidene fluoride (PVDF) membrane via electroblotting. Immunodetection was carried out using a rabbit anti-GFP primary antibody, followed by an HRP-conjugated goat anti-rabbit IgG secondary antibody.

### Isotopic analysis

Strain NyZ550, strain NyZ550Δ*metT,* and strain NyZ550Δ*metT*[pBB-MetT] were cultured in 100 mL 1/5 TSB containing the appropriate antibiotics to an OD_600 nm_ value of 0.6. The cells were harvested by centrifugation at 4,000 × *g* for 10 min at 4°C, washed three times with MSM, and then resuspended in fresh MSM to an OD_600 nm_ of 0.8 for subsequent experiments. [^13^C,^15^N]-labeled metformin was added to the cell suspension to a final concentration of 1 mM, and then the reaction mixtures were incubated at 30°C. Samples (2 mL) were collected at appropriate time intervals, centrifuged at 4,000 × *g* for 10 min at 4°C, and washed three times with PBS (pH 7.4) to remove residual [^13^C,^15^N]-labeled metformin from the cell surface. The washed cell pellets were subsequently freeze-dried and subjected to isotopic analysis.

Isotopic analysis was conducted using a Vario EL III automated elemental analyzer (Elementar Analysensysteme Comp., Hanau, Germany). The dried cell samples were first subjected to total combustion to convert the organic carbon into CO_2_. The resulting gaseous components were then introduced into the isotope ratio mass spectrometer, where they were ionized and separated based on their mass-to-charge ratios. To ensure high-precision measurements, a multi-Faraday collector system was employed to simultaneously detect ion beams corresponding to *m*/*z* 44, 45, and 46. This simultaneous collection of different mass ions enables the precise determination of the isotopic ratios by eliminating fluctuations in ion beam intensity during analysis. All isotopic measurements were calibrated against the Vienna PeeDee Belemnite international standard and normalized following established protocols (63).

### Uptake assays

The uptakes of ^14^C-labeled metformin into the cells were performed by a method described previously with minor modifications (32). The strain NyZ550Δ*metT*Δ*metCa*[pBB-MetT] and strain NyZ550Δ*metT*Δ*metCa* were grown at 30℃, 220 rpm, in 100 mL 1/5 TSB to an OD_600 nm_ value of 0.6. Cell pellets were harvested by centrifugation at 4,000 × *g* for 15 min at 4℃. The cell pellets were washed three times with 5 mM MES pH 6.5, 150 mM KCl, and resuspended in 5 mM MES, pH 6.5, 150 mM NaCl to give a final concentration of OD_600 nm_ value of 2.0. The reaction was initiated by the addition of ^14^C-metformin at a final concentration of 10 μM. An aliquot of the reaction mixture was taken at time intervals of 0, 15, 30, 90, and 120 s and centrifuged at 16,000 × *g* for 30 s at 4°C. The supernatants were removed, and the cell pellets were washed three times with 1 mL cold stop buffer (5 mM MES pH 6.5, 150 mM KCl). The intracellular radioactivity was quantified using a liquid scintillation counter (Perkin Elmer). The uptake activity was quantified and expressed as picomoles of substrates taken up per milligram of dry cell. All measurements were performed in duplicate. To investigate the cellular uptake kinetics of metformin, a concentration gradient of ^14^C-labeled metformin ranging from 0 to 50 µM was applied. The intracellular accumulation of metformin was quantified at 30 s, a time point selected based on preliminary observations indicating the uptake was near saturation.

The intracellular metformin content was quantified using a method based on UPLC-TOF-MS, as described above. The strain NyZ550Δ*metT*Δ*metCa* carrying plasmid pBB-MetT and its derived mutants were cultured as aforementioned. For uptake assays, cells were exposed to 50 μM metformin for 30 s, then immediately collected by centrifugation. After careful removal of the supernatant, cell pellets were washed three times with ice-cold PBS buffer (pH 7.4) to remove extracellular metformin, followed by lysis in an equal volume of acetonitrile to extract intracellular metformin. The lysates were then subjected to UPLC-TOF-MS analysis for metformin quantification.

### Construction of phylogenetic tree and SSN

The amino acid sequence of MetT was utilized as a query to perform a homology search against the UniProt KnowledgeBase (http://www.uniprot.org/) using the BLASTP algorithm, with a sequence identity threshold set at >30% to identify potential homologous proteins. CD-HIT was used to remove the redundant sequences with an identity threshold of 90%, which yields 85 hits. The proteins of the NCS1 family derived from bacterial sources that have been experimentally verified were manually selected. A total of 92 sequences were obtained and aligned with MAFFT, phylogenetic trees were reconstructed with IQ-TREE 2 (64) and visualized with tvBOT (65). The EFI-Enzyme Similarity Tool (EFI-EST) (https://efi.igb.illinois.edu/efi-est/) was employed to construct SSNs for MetT homologs. A total of 92 sequences were submitted to EFI-EST with an alignment score cutoff set at 120, and the resulting network visualizations were generated using Cytoscape.

### Structural prediction, molecular docking, and site-directed mutagenesis

The structure of MetT was predicted by AlphaFold3 (43), and the substrate metformin was subsequently docked into the predicted binding pocket using AutoDock Vina (v1.2.0) (44, 45). The docking conformation with the highest binding energy (−6.6 kcal/mol) was selected for further analysis and visualized using ChimeraX (66). Based on the docking results, putative substrate-binding residues were targeted for site-directed mutagenesis via PCR, using the pBB-MetT plasmid as the template. The resulting mutant plasmids were transformed into NyZ550Δ*metT*Δ*metCa* for functional characterization. Student’s t-test analysis was used to assess the statistical significance of differences in transport activity between each mutant and the wild-type MetT.

## Data Availability

All data used in this study were retrieved from accessible databases. The *metT* sequence from *Aminobacter* sp. strain NyZ550 has been deposited in GenBank (accession no. WAX94664). Sequences of the homologs MetT-7050 and MetT-1565 were retrieved from the NCBI database under accession numbers WP_183263617 and WP_092864483, respectively.

## SUPPLEMENTAL MATERIAL

Supplemental figures aem.02306-25-s0001.docx Figures S1 to S13.

ASM does not own the copyrights to Supplemental Material that may be linked to, or accessed through, an article. The authors have granted ASM a non-exclusive, world-wide license to publish the Supplemental Material files. Please contact the corresponding author directly for reuse.

## References

[1] Orive G, Lertxundi U, Brodin T, Manning P. 2022. Greening the pharmacy. Science 377:259–260. doi:10.1126/science.abp9554 35857602

[2] Bamfo NO, Hosey-Cojocari C, Benet LZ, Remsberg CM. 2021. Examination of urinary excretion of unchanged drug in humans and preclinical animal models: increasing the predictability of poor metabolism in humans. Pharm Res 38:1139–1156. doi:10.1007/s11095-021-03076-y 34254223 PMC9855226

[3] Wilkinson JL, Boxall ABA, Kolpin DW, Leung KMY, Lai RWS, Galbán-Malagón C, Adell AD, Mondon J, Metian M, Marchant RA, et al.. 2022. Pharmaceutical pollution of the world’s rivers. Proc Natl Acad Sci USA 119:11232–11238. doi:10.1073/pnas.2113947119 PMC8872717 35165193

[4] Markiewicz M, Jungnickel C, Stolte S, Białk-Bielińska A, Kumirska J, Mrozik W. 2017. Ultimate biodegradability and ecotoxicity of orally administered antidiabetic drugs. J Hazard Mater 333:154–161. doi:10.1016/j.jhazmat.2017.03.030 28349868

[5] Fujita M, Mori K, Hara H, Hishiyama S, Kamimura N, Masai E. 2019. A TonB-dependent receptor constitutes the outer membrane transport system for a lignin-derived aromatic compound. Commun Biol 2:432. doi:10.1038/s42003-019-0676-z 31799434 PMC6874591

[6] Martinez-Vaz BM, Dodge AG, Lucero RM, Stockbridge RB, Robinson AA, Tassoulas LJ, Wackett LP. 2022. Wastewater bacteria remediating the pharmaceutical metformin: genomes, plasmids and products. Front Bioeng Biotechnol 10:1086261. doi:10.3389/fbioe.2022.1086261 36588930 PMC9800807

[7] Bailey CJ. 2017. Metformin: historical overview. Diabetologia 60:1566–1576. doi:10.1007/s00125-017-4318-z 28776081

[8] Coll AP, Chen M, Taskar P, Rimmington D, Patel S, Tadross JA, Cimino I, Yang M, Welsh P, Virtue S, et al.. 2020. GDF15 mediates the effects of metformin on body weight and energy balance. Nature 578:444–448. doi:10.1038/s41586-019-1911-y 31875646 PMC7234839

[9] Yang Y, Lu X, Liu N, Ma S, Zhang H, Zhang Z, Yang K, Jiang M, Zheng Z, Qiao Y, et al.. 2024. Metformin decelerates aging clock in male monkeys. Cell 187:6358–6378. doi:10.1016/j.cell.2024.08.021 39270656

[10] Nishida M, Yamashita N, Ogawa T, Koseki K, Warabi E, Ohue T, Komatsu M, Matsushita H, Kakimi K, Kawakami E, Shiroguchi K, Udono H. 2021. Mitochondrial reactive oxygen species trigger metformin-dependent antitumor immunity via activation of Nrf2/mTORC1/p62 axis in tumor-infiltrating CD8T lymphocytes. J Immunother Cancer 9:e002954. doi:10.1136/jitc-2021-002954 34531248 PMC8449974

[11] He L, Wondisford FE. 2015. Metformin action: concentrations matter. Cell Metab 21:159–162. doi:10.1016/j.cmet.2015.01.003 25651170

[12] Graham GG, Punt J, Arora M, Day RO, Doogue MP, Duong JK, Furlong TJ, Greenfield JR, Greenup LC, Kirkpatrick CM, Ray JE, Timmins P, Williams KM. 2011. Clinical pharmacokinetics of metformin. Clin Pharmacokinet 50:81–98. doi:10.2165/11534750-000000000-00000 21241070

[13] Boldhane SP, Kuchekar BS. 2009. Gastroretentive drug delivery of metformin hydrochloride: formulation and in vitro evaluation using 3(2) full factorial design. Curr Drug Deliv 6:477–485. doi:10.2174/156720109789941641 19863493

[14] Nigam SK. 2018. The SLC22 transporter family: a paradigm for the impact of drug transporters on metabolic pathways, signaling, and disease. Annu Rev Pharmacol Toxicol 58:663–687. doi:10.1146/annurev-pharmtox-010617-052713 29309257 PMC6225997

[15] Zhou M, Xia L, Wang J. 2007. Metformin transport by a newly cloned proton-stimulated organic cation transporter (plasma membrane monoamine transporter) expressed in human intestine. Drug Metab Dispos 35:1956–1962. doi:10.1124/dmd.107.015495 17600084 PMC2672958

[16] Tanihara Y, Masuda S, Sato T, Katsura T, Ogawa O, Inui K-I. 2007. Substrate specificity of MATE1 and MATE2-K, human multidrug and toxin extrusions/H^+^-organic cation antiporters. Biochem Pharmacol 74:359–371. doi:10.1016/j.bcp.2007.04.010 17509534

[17] Sundelin E, Gormsen LC, Jensen JB, Vendelbo MH, Jakobsen S, Munk OL, Christensen M, Brøsen K, Frøkiaer J, Jessen N. 2017. Genetic polymorphisms in organic cation transporter 1 attenuates hepatic metformin exposure in humans. Clin Pharmacol Ther 102:841–848. doi:10.1002/cpt.701 28380657

[18] Shu Y, Sheardown SA, Brown C, Owen RP, Zhang S, Castro RA, Ianculescu AG, Yue L, Lo JC, Burchard EG, Brett CM, Giacomini KM. 2007. Effect of genetic variation in the organic cation transporter 1 (OCT1) on metformin action. J Clin Invest 117:1422–1431. doi:10.1172/JCI30558 17476361 PMC1857259

[19] Wu H, Esteve E, Tremaroli V, Khan MT, Caesar R, Mannerås-Holm L, Ståhlman M, Olsson LM, Serino M, Planas-Fèlix M, Xifra G, Mercader JM, Torrents D, Burcelin R, Ricart W, Perkins R, Fernàndez-Real JM, Bäckhed F. 2017. Metformin alters the gut microbiome of individuals with treatment-naive type 2 diabetes, contributing to the therapeutic effects of the drug. Nat Med 23:850–858. doi:10.1038/nm.4345 28530702

[20] Zhang R, He Y, Yao L, Chen J, Zhu S, Rao X, Tang P, You J, Hua G, Zhang L, Ju F, Wu L. 2021. Metformin chlorination byproducts in drinking water exhibit marked toxicities of a potential health concern. Environ Int 146:106244. doi:10.1016/j.envint.2020.106244 33157379

[21] Li T, Xu Z-J, Zhang S-T, Xu J, Pan P, Zhou N-Y. 2024. Discovery of a Ni^2+^-dependent heterohexameric metformin hydrolase. Nat Commun 15. doi:10.1038/s41467-024-50409-7 PMC11271267 39033196

[22] Tassoulas LJ, Rankin JA, Elias MH, Wackett LP. 2024. Dinickel enzyme evolved to metabolize the pharmaceutical metformin and its implications for wastewater and human microbiomes. Proc Natl Acad Sci USA 121:e2312652121. doi:10.1073/pnas.2312652121 38408229 PMC10927577

[23] Sinn M, Riede L, Fleming JR, Funck D, Lutz H, Bachmann A, Mayans O, Hartig JS. 2024. Metformin hydrolase is a recently evolved nickel-dependent heteromeric ureohydrolase. Nat Commun 15:8045. doi:10.1038/s41467-024-51752-5 39271653 PMC11399263

[24] Dong L, Li S, Huang J, Li W-J, Ali M. 2024. Co-occurrence, toxicity, and biotransformation pathways of metformin and its intermediate product guanylurea: current state and future prospects for enhanced biodegradation strategy. Sci Total Environ 921:171108. doi:10.1016/j.scitotenv.2024.171108 38395159

[25] de Koning H, Diallinas G. 2000. Nucleobase transporters (review). Mol Membr Biol 17:75–94. doi:10.1080/09687680050117101 10989458

[26] Patching SG. 2018. Recent developments in nucleobase cation symporter-1 (NCS1) family transport proteins from bacteria, archaea, fungi and plants. J Biosci 43:797–815. 30207323

[27] Abramson J, Wright EM. 2009. Structure and function of Na^+^-symporters with inverted repeats. Curr Opin Struct Biol 19:425–432. doi:10.1016/j.sbi.2009.06.002 19631523 PMC3496787

[28] Suzuki S, Henderson PJF. 2006. The hydantoin transport protein from Microbacterium liquefaciens. J Bacteriol 188:3329–3336. doi:10.1128/JB.188.9.3329-3336.2006 16621827 PMC1447452

[29] Weyand S, Shimamura T, Yajima S, Suzuki S, Mirza O, Krusong K, Carpenter EP, Rutherford NG, Hadden JM, O’Reilly J, Ma P, Saidijam M, Patching SG, Hope RJ, Norbertczak HT, Roach PCJ, Iwata S, Henderson PJF, Cameron AD. 2008. Structure and molecular mechanism of a nucleobase-cation-symport-1 family transporter. Science 322:709–713. doi:10.1126/science.1164440 18927357 PMC2885439

[30] Shimamura T, Weyand S, Beckstein O, Rutherford NG, Hadden JM, Sharples D, Sansom MSP, Iwata S, Henderson PJF, Cameron AD. 2010. Molecular basis of alternating access membrane transport by the sodium-hydantoin transporter Mhp1. Science 328:470–473. doi:10.1126/science.1186303 20413494 PMC2885435

[31] Simmons KJ, Jackson SM, Brueckner F, Patching SG, Beckstein O, Ivanova E, Geng T, Weyand S, Drew D, Lanigan J, Sharples DJ, Sansom MSP, Iwata S, Fishwick CWG, Johnson AP, Cameron AD, Henderson PJF. 2014. Molecular mechanism of ligand recognition by membrane transport protein, Mhp1. EMBO J 33:1831–1844. doi:10.15252/embj.201387557 24952894 PMC4195764

[32] Hatton CE, Brotherton DH, Spencer M, Cameron AD. 2022. Structure of cytosine transport protein CodB provides insight into nucleobase-cation symporter 1 mechanism. EMBO J 41:e110527. doi:10.15252/embj.2021110527 35775318 PMC9379551

[33] Ma P, Patching SG, Ivanova E, Baldwin JM, Sharples D, Baldwin SA, Henderson PJF. 2016. Allantoin transport protein, PucI, from Bacillus subtilis: evolutionary relationships, amplified expression, activity and specificity. Microbiology (Reading) 162:823–836. doi:10.1099/mic.0.000266 26967546 PMC4851255

[34] Danielsen S, Kilstrup M, Barilla K, Jochimsen B, Neuhard J. 1992. Characterization of the Escherichia coli codBA operon encoding cytosine permease and cytosine deaminase. Mol Microbiol 6:1335–1344. doi:10.1111/j.1365-2958.1992.tb00854.x 1640834

[35] Kennelly C, Prindle A. 2024. Substrate identification of putative NCS1 and NCS2 nucleobase transporters in Pseudomonas aeruginosa. mBio 15:e0243424. doi:10.1128/mbio.02434-24 39475230 PMC11633122

[36] O’Leary NA, Wright MW, Brister JR, Ciufo S, Haddad D, McVeigh R, Rajput B, Robbertse B, Smith-White B, Ako-Adjei D, et al.. 2016. Reference sequence (RefSeq) database at NCBI: current status, taxonomic expansion, and functional annotation. Nucleic Acids Res 44:D733–D745. doi:10.1093/nar/gkv1189 26553804 PMC4702849

[37] Krogh A, Larsson B, von Heijne G, Sonnhammer EL. 2001. Predicting transmembrane protein topology with a hidden Markov model: application to complete genomes. J Mol Biol 305:567–580. doi:10.1006/jmbi.2000.4315 11152613

[38] Bernsel A, Viklund H, Hennerdal A, Elofsson A. 2009. TOPCONS: consensus prediction of membrane protein topology. Nucleic Acids Res 37:W465–W468. doi:10.1093/nar/gkp363 19429891 PMC2703981

[39] Romantsov T, Battle AR, Hendel JL, Martinac B, Wood JM. 2010. Protein localization in Escherichia coli cells: comparison of the cytoplasmic membrane proteins ProP, LacY, ProW, AqpZ, MscS, and MscL. J Bacteriol 192:912–924. doi:10.1128/JB.00967-09 20008071 PMC2812954

[40] Findlay HE, Rutherford NG, Henderson PJF, Booth PJ. 2010. Unfolding free energy of a two-domain transmembrane sugar transport protein. Proc Natl Acad Sci USA 107:18451–18456. doi:10.1073/pnas.1005729107 20937906 PMC2972933

[41] Henderson PJF, Patching SG. 2022. Cloning, amplified expression, functional characterisation and purification of a Pseudomonas putida NCS1 family transport protein. Int J Adv Multidiscip Res 9:127–156.

[42] Li T, Xu ZJ, Zhou NY. 2023. Aerobic degradation of the antidiabetic drug metformin by Aminobacter sp. strain NyZ550. Environ Sci Technol 57:1510–1519. doi:10.1021/acs.est.2c07669 36624085

[43] Abramson J, Adler J, Dunger J, Evans R, Green T, Pritzel A, Ronneberger O, Willmore L, Ballard AJ, Bambrick J, et al.. 2024. Accurate structure prediction of biomolecular interactions with AlphaFold 3. Nature 630:493–500. doi:10.1038/s41586-024-07487-w 38718835 PMC11168924

[44] Morris GM, Huey R, Lindstrom W, Sanner MF, Belew RK, Goodsell DS, Olson AJ. 2009. AutoDock4 and AutoDockTools4: Automated docking with selective receptor flexibility. J Comput Chem 30:2785–2791. doi:10.1002/jcc.21256 19399780 PMC2760638

[45] Eberhardt J, Santos-Martins D, Tillack AF, Forli S. 2021. AutoDock Vina 1.2.0: new docking methods, expanded force field, and python bindings. J Chem Inf Model 61:3891–3898. doi:10.1021/acs.jcim.1c00203 34278794 PMC10683950

[46] Yamashita A, Singh SK, Kawate T, Jin Y, Gouaux E. 2005. Crystal structure of a bacterial homologue of Na^+^/Cl--dependent neurotransmitter transporters. Nature 437:215–223. doi:10.1038/nature03978 16041361

[47] Zhang S, Zhu A, Kong F, Chen J, Lan B, He G, Gao K, Cheng L, Sun X, Yan C, Chen L, Liu X. 2024. Structural insights into human organic cation transporter 1 transport and inhibition. Cell Discov 10:30. doi:10.1038/s41421-024-00664-1 38485705 PMC10940649

[48] Jaunet-Lahary T, Shimamura T, Hayashi M, Nomura N, Hirasawa K, Shimizu T, Yamashita M, Tsutsumi N, Suehiro Y, Kojima K, Sudo Y, Tamura T, Iwanari H, Hamakubo T, Iwata S, Okazaki KI, Hirai T, Yamashita A. 2023. Structure and mechanism of oxalate transporter OxlT in an oxalate-degrading bacterium in the gut microbiota. Nat Commun 14:1730. doi:10.1038/s41467-023-36883-5 37012268 PMC10070484

[49] Xu Y, Chen B, Chao H, Zhou NY. 2013. mhpT encodes an active transporter involved in 3-(3-hydroxyphenyl)propionate catabolism by Escherichia coli K-12. Appl Environ Microbiol 79:6362–6368. doi:10.1128/AEM.02110-13 23934492 PMC3811207

[50] Hong H, Patel DR, Tamm LK, van den Berg B. 2006. The outer membrane protein OmpW forms an eight-stranded β-barrel with a hydrophobic channel. J Biol Chem 281:7568–7577. doi:10.1074/jbc.M512365200 16414958

[51] Liu J, Chen S, Zhao B, Li G, Ma T. 2022. A novel FadL homolog, AltL, mediates transport of long-chain alkanes and fatty acids in Acinetobacter venetianus RAG-1. Appl Environ Microbiol 88:e0129422. doi:10.1128/aem.01294-22 36169310 PMC9599521

[52] Yoshioka S, Newell PD. 2016. Disruption of de novo purine biosynthesis in Pseudomonas fluorescens Pf0-1 leads to reduced biofilm formation and a reduction in cell size of surface-attached but not planktonic cells. PeerJ 4:e1543. doi:10.7717/peerj.1543 26788425 PMC4715448

[53] Sioupouli G, Lambrinidis G, Mikros E, Amillis S, Diallinas G. 2017. Cryptic purine transporters in Aspergillus nidulans reveal the role of specific residues in the evolution of specificity in the NCS1 family. Mol Microbiol 103:319–332. doi:10.1111/mmi.13559 27741561

[54] Witz S, Jung B, Fürst S, Möhlmann T. 2012. De novo pyrimidine nucleotide synthesis mainly occurs outside of plastids, but a previously undiscovered nucleobase importer provides substrates for the essential salvage pathway in Arabidopsis. Plant Cell 24:1549–1559. doi:10.1105/tpc.112.096743 22474184 PMC3398563

[55] Liang X, Giacomini KM. 2017. Transporters involved in metformin pharmacokinetics and treatment response. J Pharm Sci 106:2245–2250. doi:10.1016/j.xphs.2017.04.078 28495567

[56] Koepsell H. 2020. Organic cation transporters in health and disease. Pharmacol Rev 72:253–319. doi:10.1124/pr.118.015578 31852803

[57] Clifton BE, Alcolombri U, Uechi GI, Jackson CJ, Laurino P. 2024. The ultra-high affinity transport proteins of ubiquitous marine bacteria. Nature 634:721–728. doi:10.1038/s41586-024-07924-w 39261732 PMC11485210

[58] Hoang TT, Karkhoff-Schweizer RR, Kutchma AJ, Schweizer HP. 1998. A broad-host-range Flp-FRT recombination system for site-specific excision of chromosomally-located DNA sequences: application for isolation of unmarked Pseudomonas aeruginosa mutants. Gene 212:77–86. doi:10.1016/s0378-1119(98)00130-9 9661666

[59] Kovach ME, Elzer PH, Steven Hill D, Robertson GT, Farris MA, Roop RM, Peterson KM. 1995. Four new derivatives of the broad-host-range cloning vector pBBR1MCS, carrying different antibiotic-resistance cassettes. Gene 166:175–176. doi:10.1016/0378-1119(95)00584-1 8529885

[60] Stark MJR. 1987. Multicopy expression vectors carrying the lac repressor gene for regulated high-level expression of genes in Escherichia coli. Gene 51:255–267. doi:10.1016/0378-1119(87)90314-3 3110013

[61] Li S, Sun K, Yan X, Lu C, Waigi MG, Liu J, Ling W. 2021. Identification of novel catabolic genes involved in 17β-estradiol degradation by Novosphingobium sp. ES2-1. Environ Microbiol 23:2550–2563. doi:10.1111/1462-2920.15475 33754450

[62] Feilmeier BJ, Iseminger G, Schroeder D, Webber H, Phillips GJ. 2000. Green fluorescent protein functions as a reporter for protein localization in Escherichia coli. J Bacteriol 182:4068–4076. doi:10.1128/JB.182.14.4068-4076.2000 10869087 PMC94594

[63] Coplen TB, Brand WA, Gehre M, Gröning M, Meijer HAJ, Toman B, Verkouteren RM. 2006. New guidelines for δ13C measurements. Anal Chem 78:2439–2441. doi:10.1021/ac052027c 16579631

[64] Minh BQ, Schmidt HA, Chernomor O, Schrempf D, Woodhams MD, von Haeseler A, Lanfear R. 2020. IQ-TREE 2: new models and efficient methods for phylogenetic inference in the genomic era. Mol Biol Evol 37:1530–1534. doi:10.1093/molbev/msaa015 32011700 PMC7182206

[65] Xie J, Chen Y, Cai G, Cai R, Hu Z, Wang H. 2023. Tree Visualization By One Table (tvBOT): a web application for visualizing, modifying and annotating phylogenetic trees. Nucleic Acids Res 51:W587–W592. doi:10.1093/nar/gkad359 37144476 PMC10320113

[66] Pettersen EF, Goddard TD, Huang CC, Meng EC, Couch GS, Croll TI, Morris JH, Ferrin TE. 2021. UCSF ChimeraX: Structure visualization for researchers, educators, and developers. Protein Sci 30:70–82. doi:10.1002/pro.3943 32881101 PMC7737788

