# Supplementary material for: Identification of a bacterial NCS1 family transporter enabling high-affinity uptake of the antidiabetic drug metformin
Source: Appl Environ Microbiol. 2026 Jun 12;92(7):e02306-25. doi: 10.1128/aem.02306-25 (PMC13390362; doi:10.1128/aem.02306-25)
Supplement: Supplemental figures — Figures S1 to S13. [file aem.02306-25-s0001.docx]

**Supplementary materials for**

**Identification of a bacterial NCS1 family transporter enabling high-affinity uptake of the antidiabetic drug metformin**

Zhi-Jing Xu,^a^ Tao Li, ^a#^ Ning-Yi Zhou^a#^

^a^State Key Laboratory of Microbial Metabolism, Joint International Research Laboratory of Metabolic and Developmental Sciences, and School of Life Sciences and Biotechnology, Shanghai Jiao Tong University, Shanghai, China

^#^Correspondence to Ning-Yi Zhou, Email: ningyi.zhou@sjtu.edu.cn; Tao Li, Email: lisuitao@sjtu.edu.cn.

**Contents**

**Supplementary Figures**

**Supplementary Fig. 1** A conserved gene cluster is present in identified metformin degraders.

**Supplementary Fig. 2** Induction of *metT* expression by metformin treatment.

**Supplementary Fig. 3** The growth curve of strain NyZ550 and its derivatives on a range of metformin concentrations.

**Supplementary Fig. 4** Growth of strain NyZ550 and its derivatives on 10 mM glucose as sole carbon source.

**Supplementary Fig. 5** Identification of guanylurea in whole cell transformation and crude enzyme reaction.

**Supplementary Fig. 6** Membrane topology of MetT.

**Supplementary Fig. 7** Changes in δ^13^C_V-PDB_ value for strain NyZ550 and its derivatives.

**Supplementary Fig. 8** Sequence similarity network (SSN) of the MetT homologs.

**Supplementary Fig. 9** Growth of strain NyZ550 and its derivatives on a variety of purine and pyrimidine analogs.

**Supplementary Fig. 10** Structural confidence of the MetT model predicted by AlphaFold3.

**Supplementary Fig. 11** Comparison of the structures of MetT and Mhp1.

**Supplementary Fig. 12** Sequence alignment of MetT and bacterial NCS1 family transporters.

**Supplementary Fig. 13** Structural and sequence comparison of MetT and its homologs.


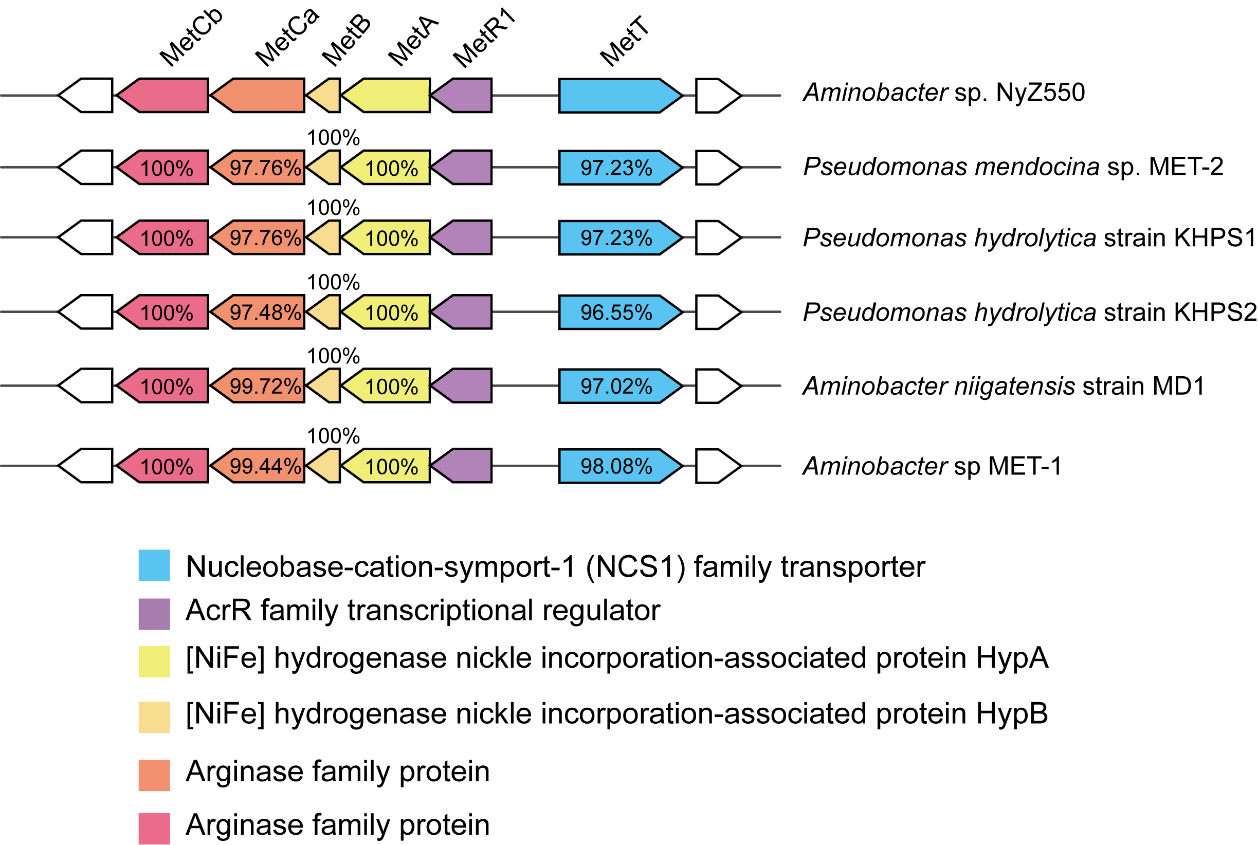


**Supplementary Fig. 1 A conserved gene cluster is present in identified metformin degraders.** The metformin utilizers include *Aminobacter* strains NyZ550, MD1 and MET-1, as well as *Pseudomonas* strains KHPS1, MET-2 and KHPS2. The gene cluster encodes six proteins, including a nucleobase cation symport 1 (NCS1) family transporter, a MerR family regulatory protein, two Ni/Fe hydrogenase nickel incorporation-associated proteins (MetA and MetB) and two arginase family proteins (MetCa and MetCb). The percentages represent the amino acid sequence identity compared to the corresponding protein sequence from strain NyZ550.


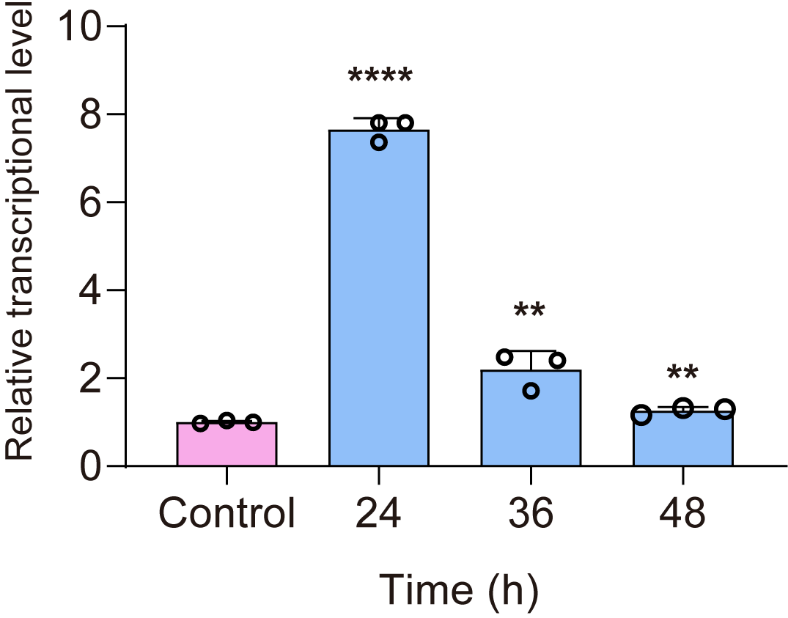


**Supplementary Fig. 2 Induction of *metT* expression by metformin treatment.** Relative mRNA levels of *metT* were measured by quantitative RT-PCR after 24, 36, and 48 hours of metformin treatment. The 16S rRNA gene of strain NyZ550 was used as an internal control for normalization. Dates are presented as the mean ± SD of three independent experiments. Statistical significance between control and treated samples at different time points was determined using Student's t-test and is denoted as ** P < 0.01, **** P < 0.0001.


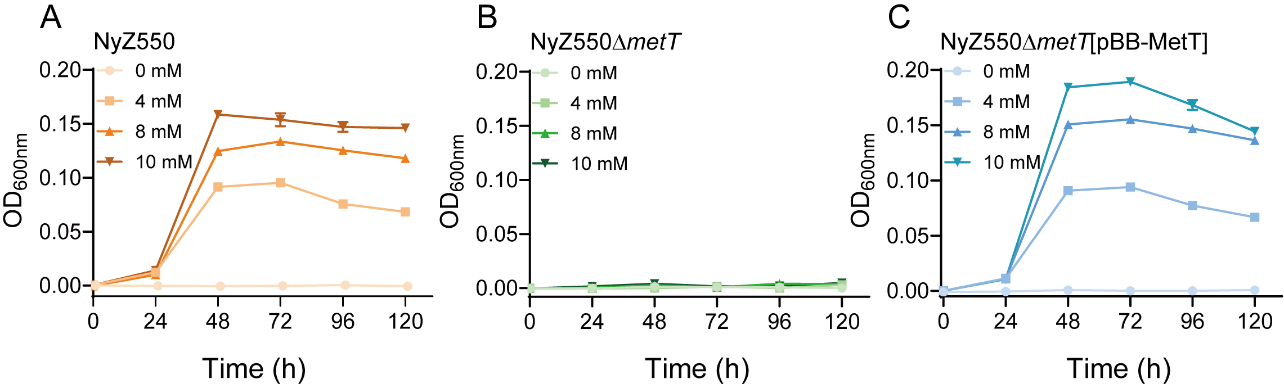


**Supplementary Fig. 3** **The growth curve of strain NyZ550 and its derivatives on a range of metformin concentrations.** Metformin (0-10 mM) was used as sole source of carbon, nitrogen and energy for growth. Values are presented as the mean ± SD of three independent experiments.


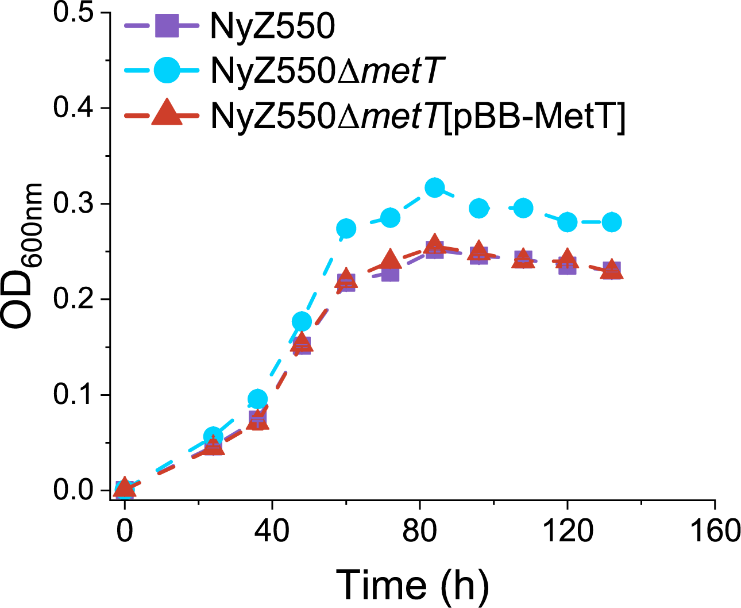


**Supplementary Fig. 4 Growth of strain NyZ550 and its derivatives on 10 mM glucose as sole carbon source.** The growth assays were performed in minimal salt media supplied with 2 mM NH_4_NO_3_ as nitrogen source. Results represent the mean ± standard deviation (SD) of three independent experiments.


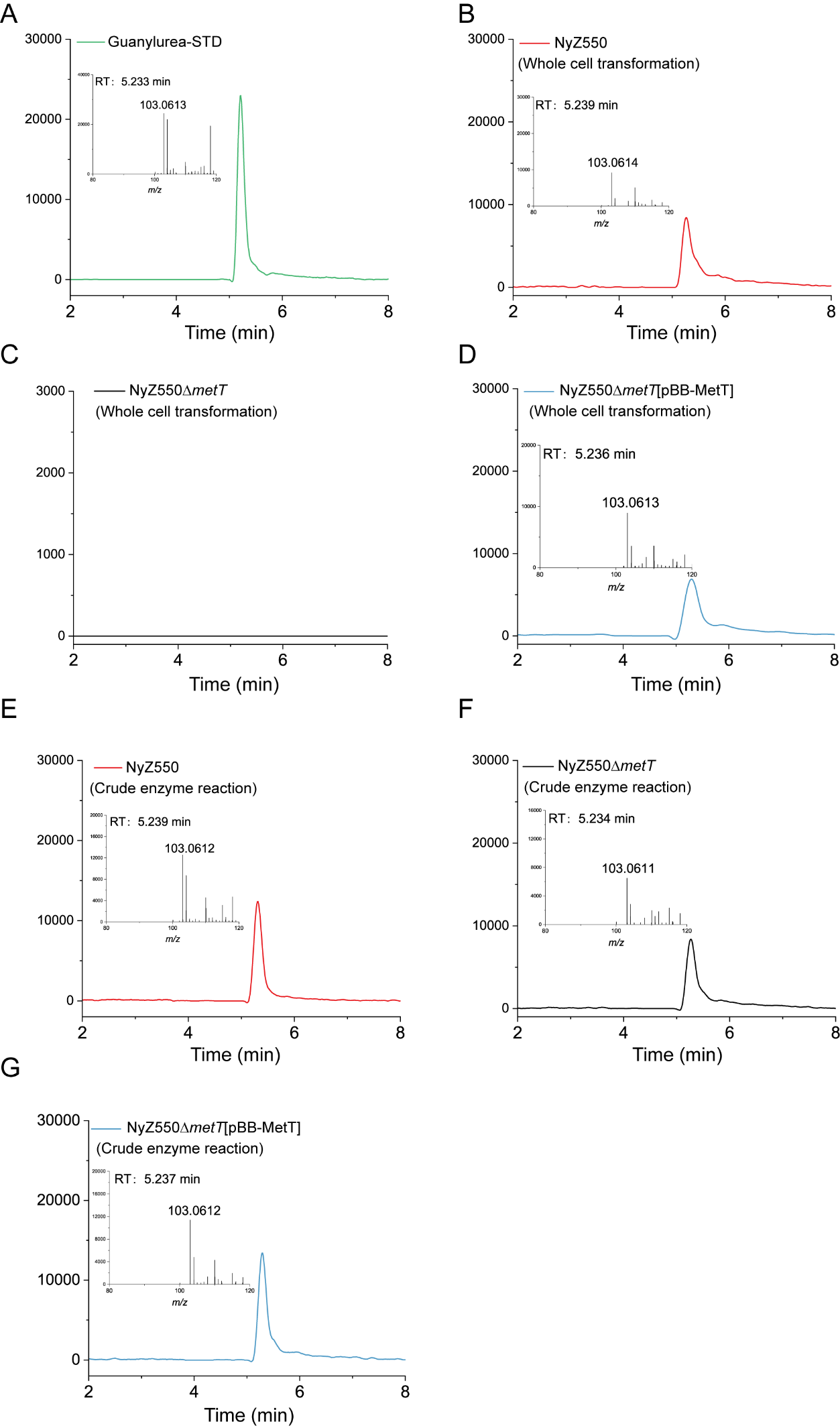


**Supplementary Fig. 5** **Identification of guanylurea in whole cell transformation and crude enzyme reaction.** UPLC-TOF-MS was used to detect the guanylurea. (A) Guanylurea standard (m/z 103.0613). (B-D) Identification of guanylurea in whole cell transformation. Samples were diluted 100 times before UPLC-TOF-MS analysis. (E-G) Identification of guanylurea in crude enzyme reaction. Samples were diluted 10 times before UPLC-TOF-MS analysis.


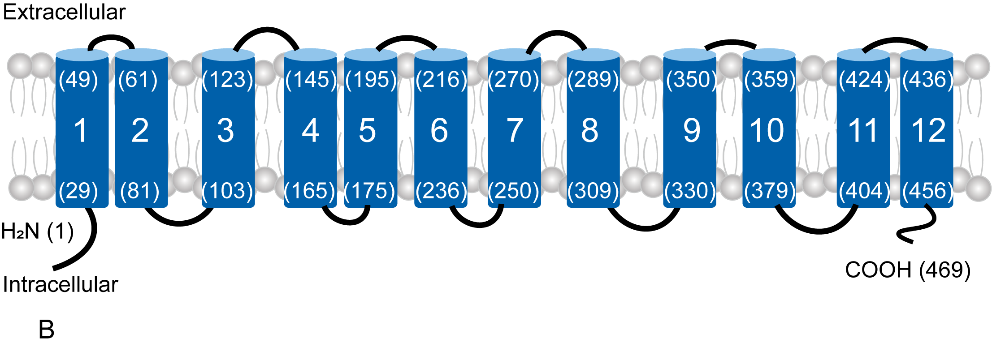


**Supplementary Fig. 6 Membrane topology of MetT.** Transmembrane analysis of MetT was performed using TOPCONS consensus prediction server (<https://topcons.cbr.su.se/pred/>).


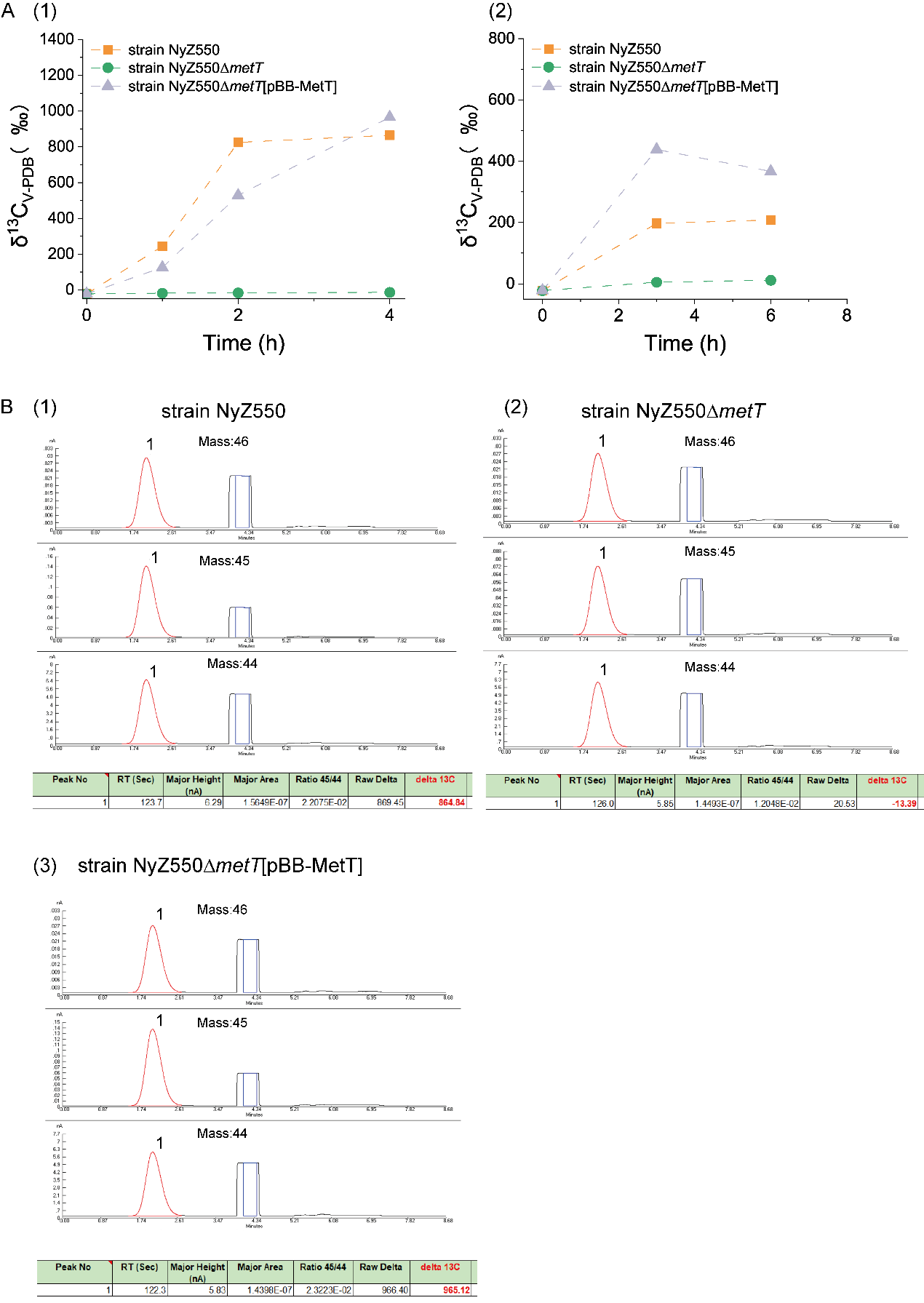


**Supplementary Fig. 7** **Transport activity of ^13^C^15^N-labelled metformin by MetT.** Data from two independent biological replicates are presented. (A) Changes in δ^13^C_V-PDB_ value of strain NyZ550 and its derivatives. (1) and (2) represent the two independent biological replicates. Strains NyZ550, NyZ550Δ*metT* and NyZ550Δ*metT*[pBB-MetT] are supplemented with 1 mM ^13^C^15^N-label metformin. Cellular samples were collected at the indicated time points and analyzed via Isotope Ratio Mass Spectrometry (IR-MS) to monitor intracellular isotopic enrichment. (B) Representative IR-MS spectra and raw data processing. The panels show the mass chromatograms (Mass 46, 45, and 44) and the calculated value of δ^13^C_V-PDB_ are marked in red.


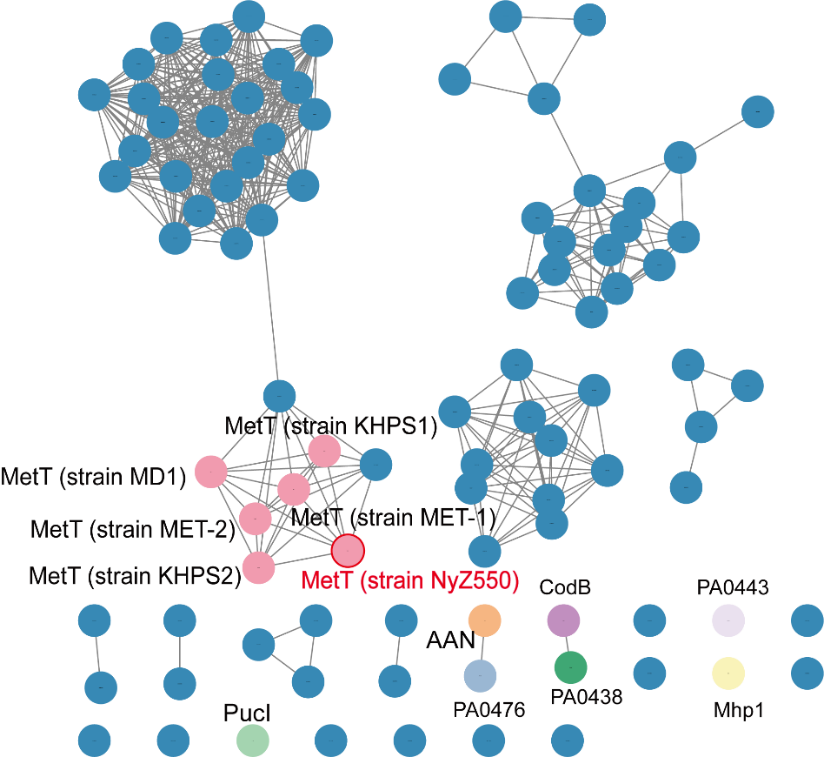


**Supplementary Fig. 8 Sequence similarity network (SSN) of the MetT homologs.** MetT (strain NyZ550): identified in this study; MetT (strain KHPS1, strain KHPS2, strain MD1, strain MET-1 and strain MET-2): MetT homologs from other metformin utilizers; Mhp1: hydantoin transporter; CodB: cytosine transporter; PucI: allantoin transporter; AAN: allantoin transporter; PA0438: cytosine and 5-fluorocytosine transporter; PA0476: allantoin transporter; PA0443: cytosine, thymine, uracil and dihydrouracil transporter.


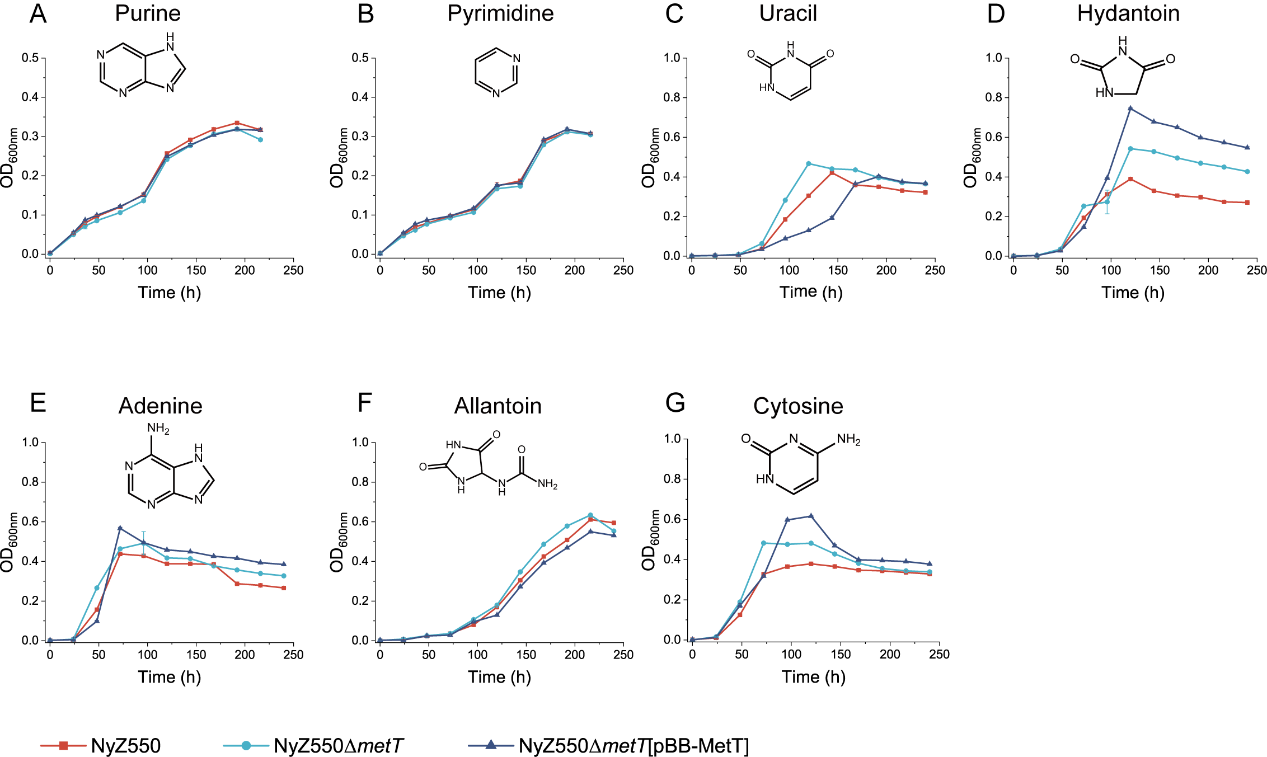


**Supplementary Fig. 9** **Growth of strain NyZ550 and its derivatives on a variety of purine and pyrimidine analogs.** Compounds (2 mM each) indicated were used as the sole source of nitrogen, supplemented with 10 mM glucose as the carbon source. All measurements were performed in triplicate, and the data are shown as mean ± SD.


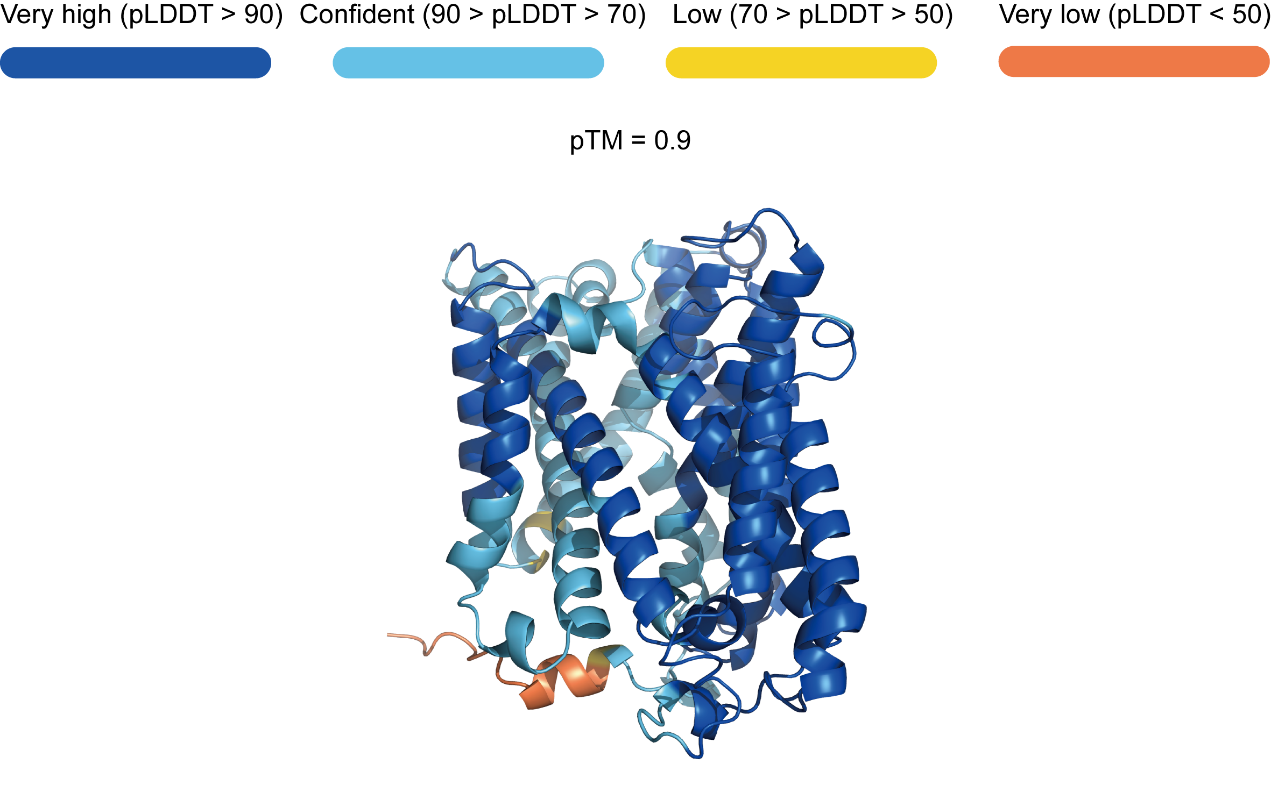


**Supplementary Fig. 10 Structural confidence of the MetT model predicted by AlphaFold3.** The full-length structural model of MetT is shown in cartoon representation. Residues are colored according to their pLDDT (predicted Local Distance Difference Test) scores, which represent the model's local confidence: blue (pLDDT > 90, very high confidence), cyan (90 > pLDDT > 70, confident), yellow (70 > pLDDT > 50, low confidence), and orange (pLDDT < 50, very low confidence). The core transmembrane helices exhibit high structural reliability (pLDDT > 90), supporting the validity of the subsequent docking and functional analyses.


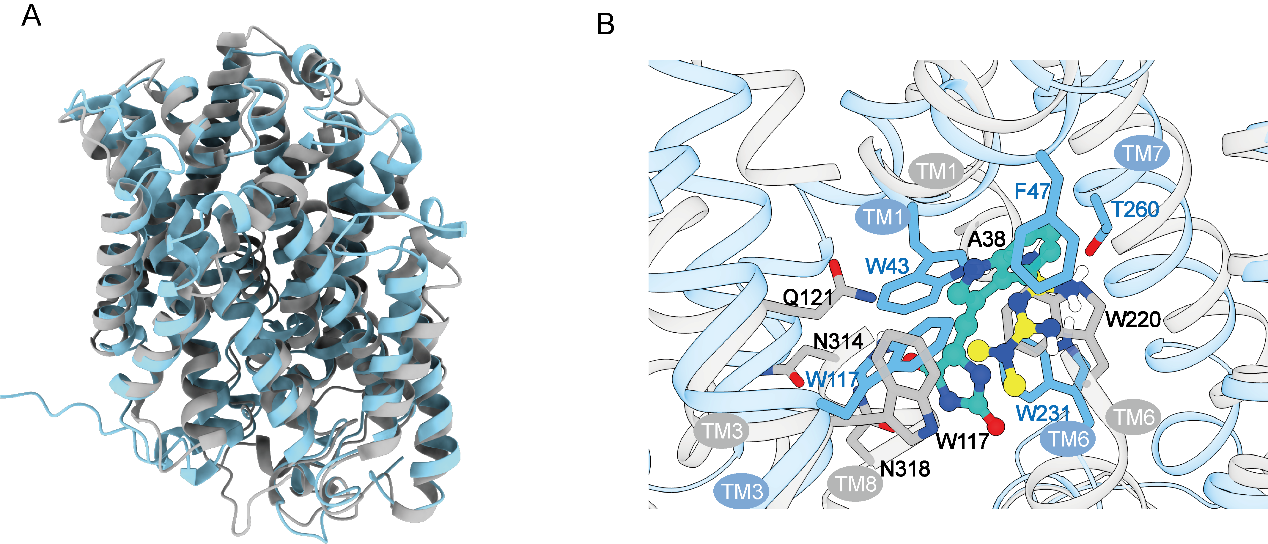


**Supplementary Fig. 11** **Comparison of the structures of MetT and Mhp1.** (A) Comparing the AlphaFold model of MetT (blue) with the inward-facing conformation of Mhp1 (PDB: 2X79, gray). The alignment yielded a Root Mean Square Deviation (RMSD) of 5.627 Å across all 437 paired atoms. (B) Detailed view of the substrate-binding pockets of MetT (blue) and substrate binding form of Mhp1 (PDB: 4D1A, gray). The transport substrates of MetT (metformin) and Mhp1 (hydantoin), are represented as yellow and green, respectively. Key transmembrane helices and the corresponding amino acid residues involved in substrate transport are highlighted and labeled in the figure.


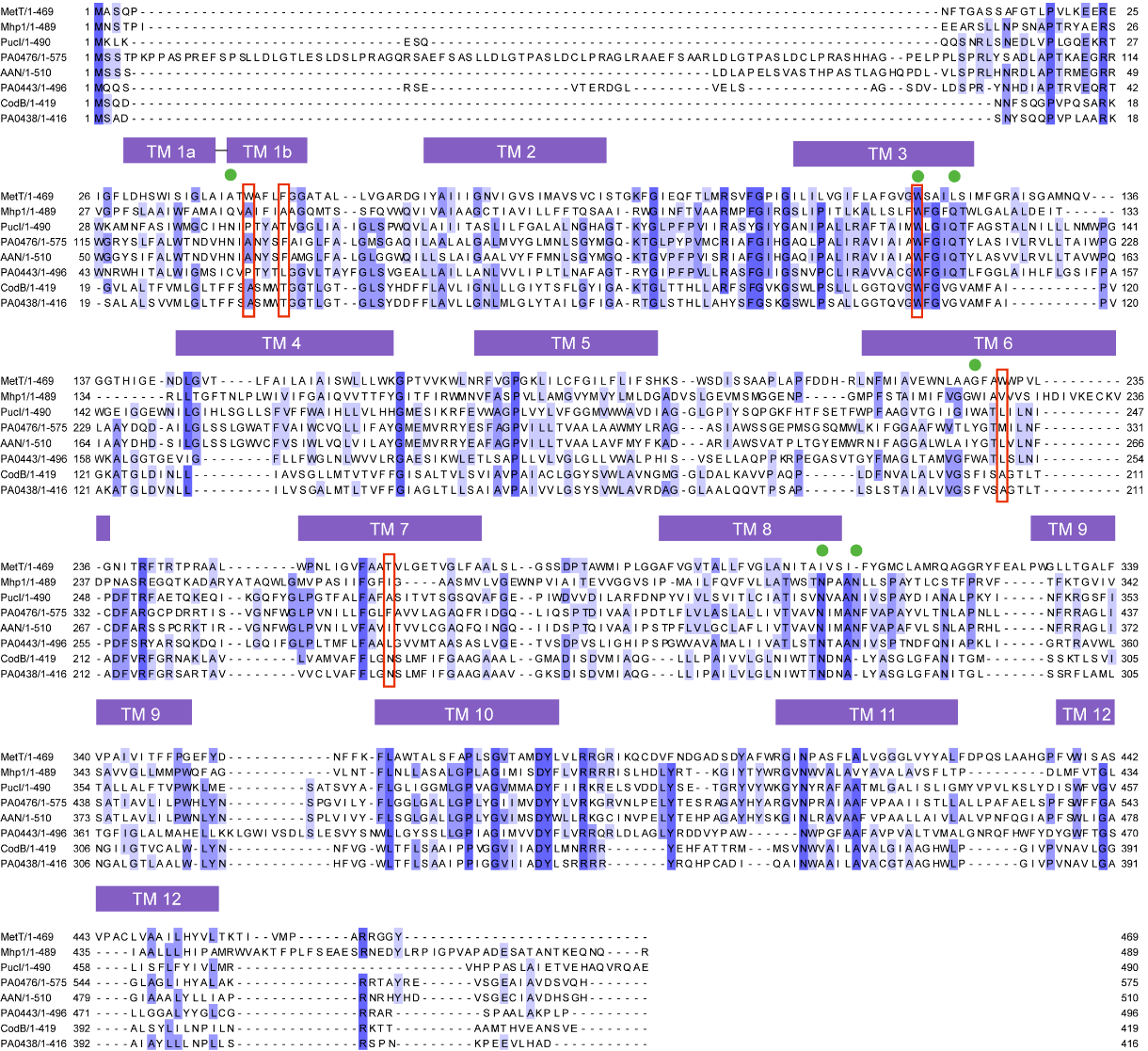


**Supplementary Fig. 12 Sequence alignment of MetT and bacterial NCS1 family transporters.** Multiple sequence alignment of MetT and representative bacterial NCS1 family transporters was performed using MAFFT. Sequence conservation is indicated by shading. The predicted secondary structure of MetT is shown above the alignment. Residues predicted to interact with the substrate are highlighted with red boxes. The amino acids involved in benzyl-hydantoin binding in Mhp1 are marked with green circles.


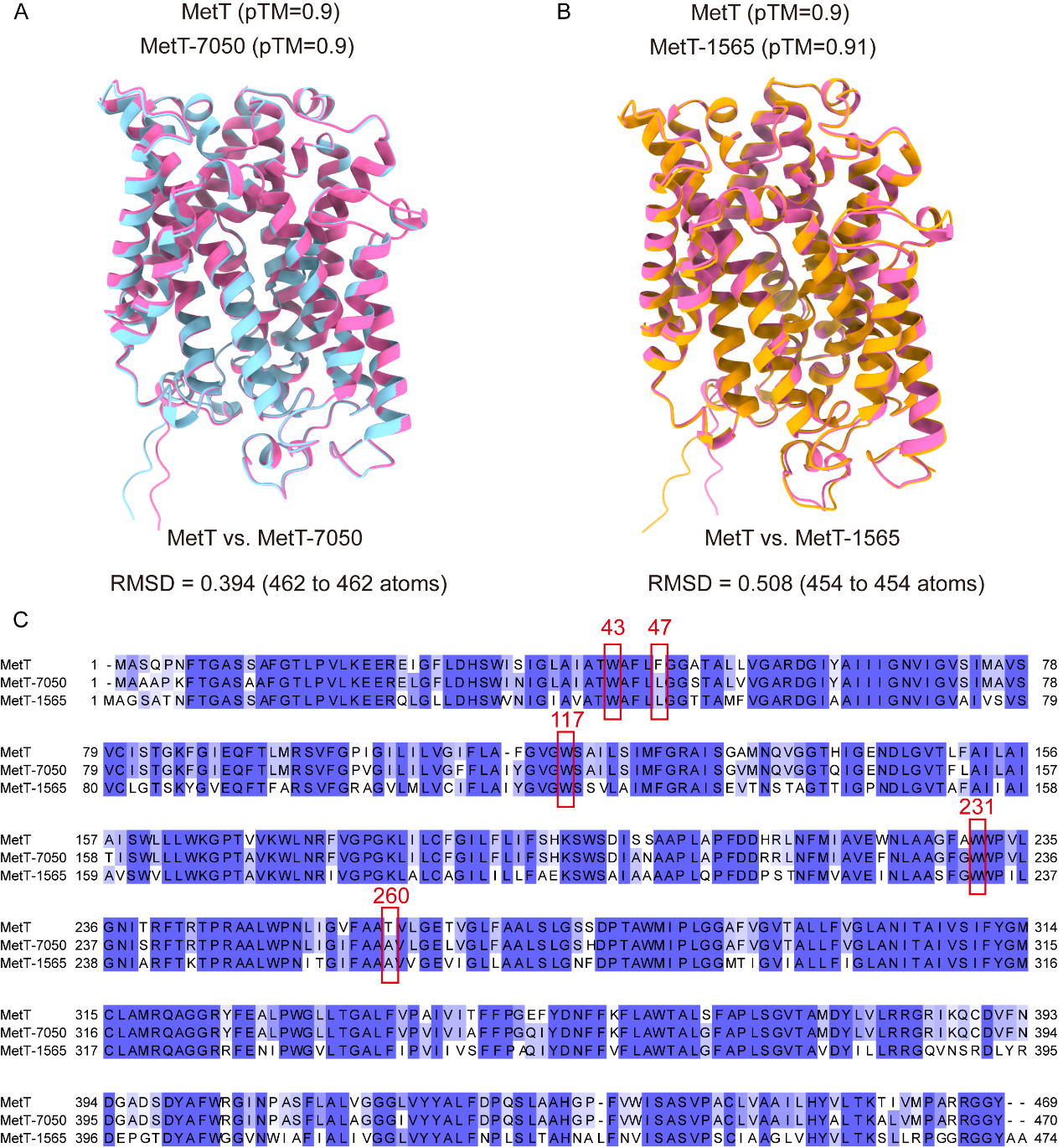


**Supplementary Fig. 13 Structural and sequence comparison of MetT and its homologs.** (A-B) Comparation of the AlphaFold3 predicted structures of MetT (pink) with its homologs MetT-7050 (light blue) and MetT-1565 (orange). The Root Mean Square Deviation (RMSD) values are indicated below each panel. A predicted template modeling (pTM) score above 0.5 indicates that the overall predicted structure is reliable. (C) Multiple sequence alignment of MetT, MetT-7050, and MetT-1565. Critical residues predicted to participate in substrate binding (W43, F47, W117, W231, and T260) are highlighted with red boxes and numbered according to the MetT sequence.
